# Diagnostic Performance of a Deep Learning Model Using Phonocardiogram and Single‐Lead ECG Signals for Detecting Moderate‐or‐Greater Aortic Stenosis: Multicenter External Validation

**DOI:** 10.1002/clc.70498

**Published:** 2026-10-15

**Authors:** Shumpei Saito, Masanobu Ishii, Shimpei Ogawa, Hiroshi Seki, Koshiro Ikeda, Youhei Kayano, Yuhei Yasui, Ginga Sato, Mitsuru Ohishi, Takuro Kubozono, Naritatsu Saito, Eri Toda Kato, Masahiro Yamada, Shunsuke Natori, Yuki Kunikane, Takafumi Yokomatsu, Masashi Kato, Yasuaki Sagara, Nami Uchiyama, Nobuhiko Atsuchi, Shota Kawahara, Shoji Natsugoe, Noriaki Tabata, Kenichi Tsujita

**Affiliations:** ^1^ AMI Inc Kagoshima Japan; ^2^ Department of Medical Information Science, Graduate School of Medical Sciences Kumamoto University Kumamoto Japan; ^3^ Department of Cardiovascular Medicine and Hypertension, Graduate School of Medical and Dental Sciences Kagoshima University Kagoshima Japan; ^4^ Department of Cardiovascular Medicine Kyoto University Hospital Kyoto Japan; ^5^ Institute for Advancement of Clinical and Translational Science Kyoto University Hospital Kyoto Japan; ^6^ Division of Cardiology Saiseikai Kumamoto Hospital Cardiovascular Center Kumamoto Japan; ^7^ Department of Cardiology Hokkaido Social Work Association Furano Hospital Hokkaido Japan; ^8^ Department of Cardiology Mitsubishi Kyoto Hospital Kyoto Japan; ^9^ Department of Breast Surgical Oncology Sagara Hospital Kagoshima Japan; ^10^ Department of Cardiology Sagara Hospital Kagoshima Japan; ^11^ Department of Cardiology Tenyoukai Central Hospital and Central Clinic Kagoshima Japan; ^12^ Kajiki Onsen Hospital Kagoshima Japan; ^13^ Department of Cardiovascular Medicine, Graduate School of Medical Sciences Kumamoto University Kumamoto Japan

**Keywords:** aortic stenosis, deep learning, heart sounds, valvular heart disease

## Abstract

**Background:**

Valvular heart disease, particularly aortic stenosis (AS), often remains asymptomatic until advanced stages, making early detection challenging. Although cardiac auscultation is a traditional screening tool, its diagnostic accuracy is limited. We aimed to externally validate a deep learning–based model (eAS model) using phonocardiogram (PCG) and single‐lead electrocardiogram (ECG) signals to detect moderate‐or‐greater AS.

**Methods:**

PCG and ECG recordings were acquired using a PCG device (Cardio‐EGG). Transthoracic echocardiography served as the reference standard. The external validation cohort consisted of 951 patients from 3 medical institutions, including 70 with moderate‐or‐greater AS. The primary outcome was sensitivity for detecting moderate‐or‐greater AS; secondary outcomes included the area under the receiver operating characteristic curve (AUROC), specificity, predictive values, and likelihood ratios (LR).

**Results:**

The external validation cohort (*n* = 951, median age 73 years, male 65%) exhibited more advanced valvular disease than the training/development cohort. The eAS model achieved an AUROC of 0.9632 (95% CI: 0.9471–0.9774). Using the pre‐determined threshold, sensitivity was 92.9% and specificity 87.9%, yielding a positive LR of 7.65 and negative LR of 0.08. Model‐derived probabilities showed strong associations with echocardiographic measures of AS severity (adjusted *R* ^2^ = 0.5051 for peak transvalvular velocity). Exploratory subgroup analyses showed generally consistent performance, although estimates were imprecise in small subgroups.

**Conclusion:**

The eAS model demonstrated high diagnostic performance for detecting moderate‐or‐greater AS in a multicenter external validation cohort. AI‐based PCG/ECG analysis may support the decision to refer patients from auscultation to echocardiography for AS evaluation.

## Practitioner Points

- An AI model using 8‐s phonocardiogram and single‐lead ECG recordings demonstrated high accuracy for detecting moderate‐or‐greater aortic stenosis in a multicenter external validation cohort.
- Model outputs were strongly associated with key echocardiographic indices of AS severity, supporting biological plausibility.
- AI‐based PCG/ECG analysis may complement conventional auscultation as a diagnostic aid for clinically significant AS in an echocardiography‐referred cardiology population.

## Graphical abstract

In an independent multicenter cohort of 951 patients undergoing echocardiography, a deep learning model analyzing an 8‐s heart sound and ECG recording detected moderate‐or‐greater aortic stenosis with 92.9% sensitivity and 87.9% specificity. This approach may support the decision to refer patients from auscultation to echocardiography.

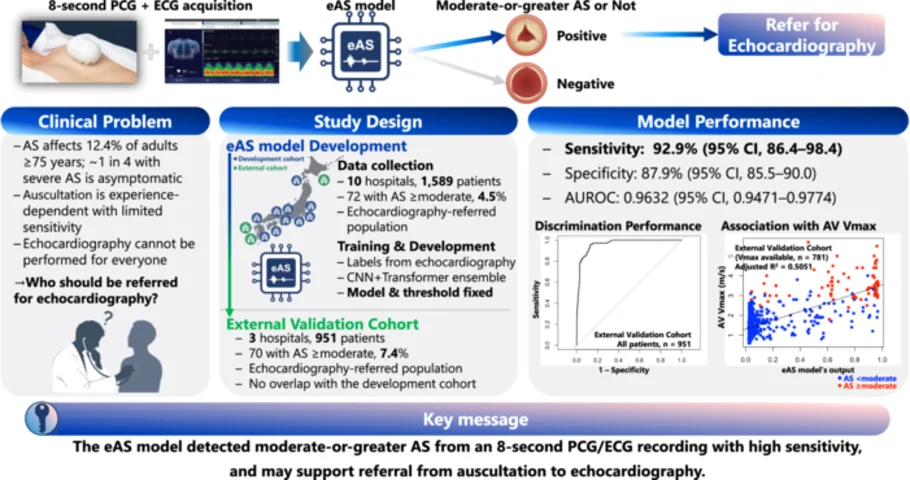

## 1 Introduction

In developed countries such as Japan, the United States, and European nations, population aging has led to an increasing prevalence of calcific aortic valve disease and degenerative mitral valve disease [1]. Among valvular diseases, aortic stenosis (AS) represents a particularly critical condition, with an estimated prevalence of 12.4% in individuals aged ≥ 75 years, including 3.4% with severe AS [2]. The prognosis of untreated symptomatic AS is extremely poor, with a reported 1‐year mortality rate of 53.1%, compared with 6.4% among patients who underwent surgical aortic valve replacement (SAVR) [3]. Approximately 25% of patients with severe AS are asymptomatic [2], and symptoms in elderly patients are often nonspecific, leading to delayed diagnosis. Even in asymptomatic cases, progression to symptom onset or death within 2–3 years is common [4, 5], and recent evidence suggests that early intervention with SAVR or transcatheter aortic valve replacement (TAVR) can reduce adverse outcomes [6, 7, 8, 9, 10, 11]. Accordingly, contemporary guidelines recommend early detection, close follow‐up, and timely consideration of intervention [12, 13, 14].

Despite the importance of early detection, a substantial proportion of patients remain undiagnosed in routine clinical practice [15, 16]. For example, a population‐based echocardiographic screening study in the United Kingdom reported that 11.3% of individuals aged ≥ 65 years had clinically significant valvular heart disease requiring treatment, with 6.4% being newly diagnosed cases [17]. Physical examination, particularly cardiac auscultation, has long served as a fundamental screening tool for valvular disease; however, its diagnostic performance is highly dependent on physician experience and suffers from limited reproducibility. Recent systematic reviews have highlighted its limited sensitivity and suboptimal balance with specificity, underscoring the limitations of physical examination as a stand‐alone screening method [18, 19, 20, 21].

In recent years, digital phonocardiography devices have been miniaturized and simplified, supporting broader clinical adoption. In Japan, Cardio‐EGG (AMI Inc., Kagoshima, Japan), a regulatory‐approved phonocardiography device capable of recording cardiac physiological signals within 8 s, has been introduced into clinical practice [22]. While this technology enables visualization of characteristic ejection systolic murmurs associated with AS, interpretation remains dependent on expertise, and challenges regarding reproducibility and diagnostic accuracy persist.

Advances in artificial intelligence (AI) and deep learning have opened new possibilities for screening cardiovascular diseases using noninvasive physiological signals such as phonocardiograms (PCG) and electrocardiograms (ECG). While several studies have attempted to develop models for valvular heart disease detection using PCG [23, 24], we previously reported a deep learning model that predicted cardiac overload, represented by B‐type natriuretic peptide (BNP), using PCG and ECG signals acquired by Cardio‐EGG, achieving an area under the receiver operating characteristic curve (AUROC) of 0.895 in an external validation cohort [25]. Building on this work, we developed a deep‐learning model to detect clinically significant AS of at least moderate severity. In the present study, we aimed to evaluate the diagnostic performance of this model in an independent external validation cohort.

## 2 Methods

### 2.1 Study Design and Participants

This was a multicenter, cross‐sectional observational study with prospectively collected data. The external validation cohort was designed to reflect a broad, real‐world echocardiography‐referred population and comprised two tertiary hospitals and one community general hospital. The database was not specifically designed to enroll patients with valvular heart disease or AS, but rather to include a broad range of patients undergoing echocardiography in routine clinical practice. Between February and December 2021, patients aged ≥ 18 years who provided written informed consent and underwent routine transthoracic echocardiography (TTE) were consecutively enrolled whenever feasible at each participating center. Exclusion criteria were: (1) missing PCG or ECG recordings, (2) lack of TTE‐based AS severity assessment, and (3) an interval > 6 months between PCG/ECG acquisition and TTE (Figure 1). The study protocol was approved by the institutional review boards of all participating centers and was conducted in accordance with the Declaration of Helsinki and applicable regulations.

**Figure 1:**
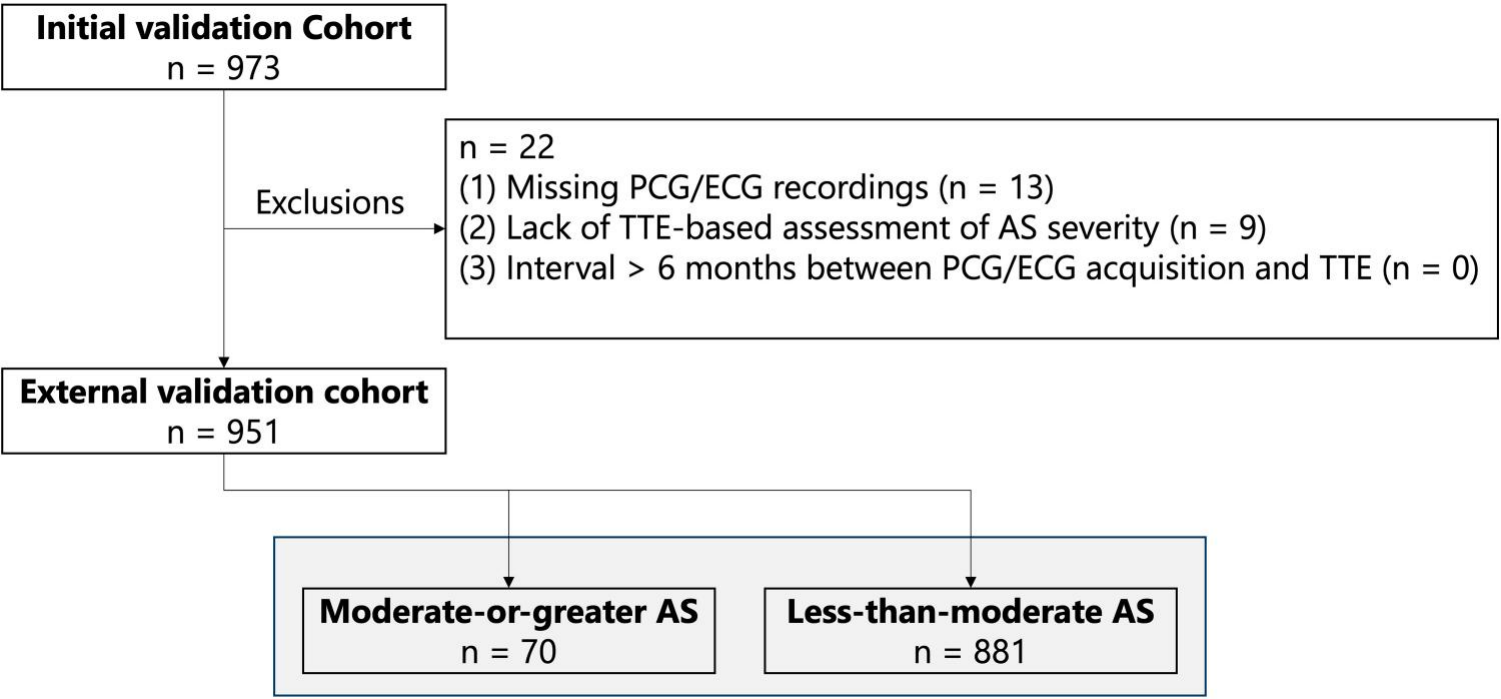
Flowchart of patient selection for external model validation**.** The initial validation cohort consisted of 973 patients. After excluding 22 patients because of missing PCG/ECG recordings (*n* = 13), lack of TTE–based assessment of AS severity (*n* = 9), or an interval longer than 6 months between PCG/ECG acquisition and TTE (*n* = 0), a total of 951 patients were included in the external validation cohort. Patients were subsequently categorized according to AS severity into moderate or greater AS (*n* = 70) and less than moderate AS (*n* = 881). AS, aortic stenosis; ECG, electrocardiogram; PCG, phonocardiogram; TTE, transthoracic echocardiography.

### 2.2 PCG and Single‐Lead ECG Data Acquisition and Preprocessing

PCG signals and synchronized single‐lead ECG data were acquired using the Cardio‐EGG device (AMI Inc., Japan) (Figure 2). Recordings were obtained in the supine position at rest, with the device placed at the fourth left sternal border (4LSB) for approximately 16 s. Because Cardio‐EGG is routinely used to obtain 8‐s recordings in clinical practice, 8‐s segment was extracted from each recording, starting 5 s after acquisition onset and including respiratory sounds. Signals were resampled at 2 kHz and amplitude‐normalized to zero mean and unit variance.

**Figure 2:**
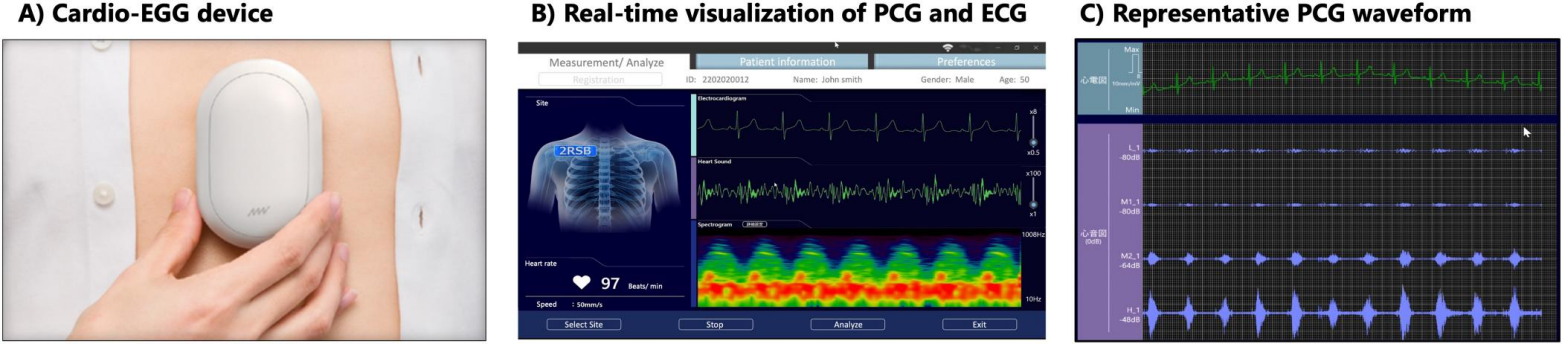
Overview of the Cardio‐EGG system for PCG and ECG acquisition and visualization. (A) Cardio‐EGG (AMI Inc., Japan) is a PCG device that enables acquisition of synchronized PCG and ECG signals within 8 s by direct placement on the precordial chest wall. (B) Real‐time visualization interface displaying ECG, heart sound waveforms, and time–frequency representations during signal acquisition. (C) Representative PCG waveform illustrating typical heart sound findings in a case of aortic stenosis. ECG, electrocardiogram; PCG, phonocardiogram.

### 2.3 Collection of Reference Standard and Clinical Data

TTE‐based assessment of AS severity served as the reference standard in both the training/development and external validation cohorts. Diagnostic criteria followed the Japanese Circulation Society guidelines on the management of valvular heart disease [14], namely: peak aortic valve velocity (Vmax) ≥ 3.0 m/s, mean transvalvular pressure gradient (mean PG) ≥ 20 mmHg, and/or aortic valve area (AVA) ≤ 1.5 cm^2^ measured by continuous‐wave Doppler. When the valve appeared grossly normal on qualitative assessment, quantitative Doppler parameters were not always separately recorded, and classification as no AS was based on the overall echocardiographic impression. Final AS severity was adjudicated by cardiologists integrating echocardiographic findings with clinical information, blinded to Cardio‐EGG recordings and the eAS model outputs. This adjudication was based on the TTE report generated as part of routine clinical care at each site; formal central reading or multi‐reader adjudication was not performed.

Baseline demographic and clinical data variables included age, sex, body mass index (BMI), and systolic blood pressure, were extracted from medical records. TTE parameters obtained within 6 months of PCG/ECG acquisition were considered valid, including systolic/diastolic cardiac function, and aortic regurgitation (AR), mitral regurgitation (MR), and tricuspid regurgitation (TR).

Plasma BNP levels measured within ±30 days of PCG/ECG acquisition were used. Atrial fibrillation (AF) was assessed using single‐lead ECG waveforms from Cardio‐EGG, independently reviewed by two cardiologists, with discrepancies resolved by consensus.

### 2.4 Model Development and Training Strategy

Deep learning models for detecting moderate‐or‐greater AS were developed using PCG and ECG signals acquired with Cardio‐EGG from multiple anatomical recording positions. The Training/Development cohort consisted of 1589 cases collected between February 2021 and July 2023 across 10 hospitals in Japan, including general hospitals, regional hospitals, and tertiary centers, and was entirely independent of the external validation cohort. Model development used stratified five‐fold cross‐validation according to AS severity with patient‐level data splitting (training set, 80%; development set, 20%), and final predictions were derived by ensembling probability outputs from the individual models. Model development followed the TRIPOD + AI reporting guideline, with full methodological details provided in the Supporting Information Methods.

The AI framework incorporated two architectures, a Convolutional Neural Network (CNN) and a Transformer, to leverage their complementary strengths. The CNN model was based on a 10‐layer SE‐ResNet [26] backbone with a self‐attention pooling mechanism, followed by fully connected layers to output probabilities between 0 and 1 for the presence of moderate‐or‐greater AS. The Transformer model was constructed on the wav2vec 2.0 framework [27], taking synchronized PCG and ECG signals as input. Local signal features were first extracted by a CNN encoder, and temporal dependencies were modeled by a Transformer encoder. The model underwent self‐supervised pretraining on unlabeled PCG/ECG data, followed by supervised fine‐tuning with labeled data, ultimately outputting probabilities for the presence of AS.

During training, short waveform segments were randomly sampled from each recording, and data augmentation techniques—including Gaussian noise addition, superposition of environmental sounds or speech, filtering, and speed perturbation—were applied to enhance input diversity (Supporting Information S1: Table 1).

The final binary output of the ensemble model was determined using a Youden Index‐based threshold derived from the development dataset and fixed at 0.148 for all analyses (Supporting Information S1: Table 2). The final model and classification threshold were locked before inference on the external validation cohort and remained unchanged throughout the validation analysis. In the external validation cohort, only recordings acquired specifically at the 4LSB were used for evaluation.

### 2.5 Statistical Analysis and Model Evaluation

After application of the exclusion criteria, the external validation cohort (*n* = 951) comprised 70 patients with moderate‐or‐greater AS and 881 controls with less‐than‐moderate AS. The primary outcome was the sensitivity for detecting moderate‐or‐greater AS. This sample size was based on estimation precision rather than a power calculation against a null hypothesis, assuming a sensitivity of 0.80 and a target 95% confidence interval (CI) half‐width of 0.10, which required at least 62 patients with moderate‐or‐greater AS and, for comparable precision in specificity, at least 62 patients without moderate‐or‐greater AS. Secondary outcomes included AUROC, specificity, positive predictive value (PPV), negative predictive value (NPV), and positive/negative likelihood ratios (LR+/LR−). The 95% CIs were estimated using a nonparametric bootstrap method with 2000 patient‐level resamples. The classification threshold was fixed based on the development cohort. Three additional analyses of discriminative performance were performed, as detailed in the Supporting Information Methods. First, to evaluate the relative contribution of each input modality, single‐modality (PCG‐only and ECG‐only) and combined‐modality models were compared using the wav2vec architecture. Second, the incremental diagnostic value of the eAS model output beyond routinely available clinical and 12‐lead ECG variables was assessed using nested logistic regression models. Third, model performance was re‐evaluated after restricting the external validation cohort to shorter intervals between PCG/ECG acquisition and echocardiography.

Continuous variables were summarized as median (interquartile range), whereas categorical variables were expressed as number (%). Associations between the AI‐derived probabilities and echocardiographic parameters (Vmax, mean PG, AVA) were assessed using univariable linear regression, with adjusted *R* ^2^ values reported.

Exploratory subgroup analyses were conducted according to age, sex, BMI, AF, moderate‐or‐greater AR, MR, TR, and left ventricular ejection fraction (LVEF) measured by the modified Simpson's method. Severe AS was further classified according to flow‐gradient hemodynamic phenotype using stroke volume index, as detailed in the Supporting Information Methods [14]. Reference‐standard uncertainty was further evaluated through sensitivity analyses, including a Monte Carlo perturbation analysis using inter‐observer variability–derived standard deviations (Supporting Information S1: Table 3; Supporting Information Methods). Model calibration was assessed using calibration plots, calibration intercept and slope, Brier score, and the observed‐to‐expected event ratio, with recalibration performed using the development cohort. Precision‐recall curves, average precision, decision curve analysis, and projected diagnostic yield across assumed AS prevalence scenarios were also calculated (Supporting Information Methods). Model development was conducted using Python version 3.9.7, and statistical analyses using R version 4.5.2. All tests were two‐tailed, and a *p* < 0.05 considered statistically significant.

Because the final eAS model output was generated by an ensemble of the CNN and Transformer components, these Gradient‐weighted Class Activation Mapping (Grad‐CAM) visualizations reflect the contribution of the CNN component alone and do not represent the ensemble prediction in its entirety.

## 3 Results

Table 1 summarizes the clinical characteristics of the training/development cohort (*n* = 1589) and the external validation cohort (*n* = 951). The prevalence of moderate‐or‐greater AS was 4.5% in the training/development cohort and 7.4% in the external validation cohort. Compared with the training/development cohort, the external validation cohort exhibited a slightly higher proportion of moderate‐or‐greater AS, a lower LVEF within the low‐normal range, and a tendency toward higher E/e' values within the high‐normal range.

**Table 1:** Clinical characteristics of the training/development and external validation cohorts.

Variables	Training/development cohort (*n* = 1589)	External validation cohort (*n* = 951)
Age, years	71 [61, 78]	73 [64, 81]
Sex, male (%)	888/1589 (55.9)	616/951 (64.8)
BMI, kg/m^2^	23.5 [21.4, 26.2]	23.2 [20.7, 25.6]
Systolic BP, mmHg	130 [116, 145]	136 [121, 152]
BNP, pg/mL	82 [28, 236]	61 [23, 174]
LVEF, %	67 [57, 74]	59 [50, 63]
E/e'	9.9 [7.8, 12.5]	11.2 [8.8, 15.1]
Aortic valve replacement (%)	61/1589 (3.8)	46/951 (4.8)
AS severity (%)		
None	1468/1589 (92.4)	838/951 (88.1)
Mild	49/1589 (3.1)	43/951 (4.5)
Moderate	36/1589 (2.3)	44/951 (4.6)
Severe	36/1589 (2.3)	26/951 (2.7)

*Note:* Values are presented as median [interquartile range] or *n*/*N* (%). Denominators reflect the number of patients with non‐missing data for each variable. The training/development cohort consisted of cases used for model development, whereas the external validation cohort was an independent cohort used exclusively for performance evaluation.

Abbreviations: AS, aortic stenosis; BMI, body mass index; BNP, B‐type natriuretic peptide; BP, blood pressure; E/e', ratio of early mitral inflow velocity to mitral annular early diastolic velocity; LVEF, left ventricular ejection fraction.

In the external validation cohort, patients with moderate‐or‐greater AS exhibited echocardiographic parameters consistent with hemodynamically significant AS, with a median Vmax of 3.4 m/s, a median mean PG of 27.0 mmHg, and a median AVA of 0.90 cm^2^. In contrast, the control group (less‐than‐moderate AS) demonstrated preserved valve opening, with corresponding values of 1.4 m/s, 4.0 mmHg, and 2.10 cm^2^, respectively (Table 2). The extent of missing data for clinical variables is summarized in Supporting Information S1: Table 4. Among patients classified as having no AS, all three quantitative severity parameters were missing in 167 of 838 patients (19.9%), whereas at least two of the three parameters were recorded in every patient classified as having mild‐or‐greater AS (Supporting Information S1: Table 5).

**Table 2:** Clinical characteristics according to aortic stenosis severity.

Variables	Overall (*n* = 951)	Moderate or greater AS (*n* = 70)	Less than moderate AS (*n* = 881)
Age, years	73 [64, 81]	82 [73, 87]	72 [63, 81]
Sex, male (%)	616/951 (64.8)	40/70 (57.1)	576/881 (65.4)
BMI, kg/m^2^	23.2 [20.7, 25.6]	22.9 [20.0, 25.8]	23.1 [20.7, 25.5]
Systolic BP, mmHg	136 [121, 152]	143 [128, 160]	135 [121, 151]
Atrial fibrillation (%)	85/853 (10.0)	4/65 (6.2)	81/788 (10.3)
BNP, pg/mL	61 [23, 174]	109 [45, 285]	58 [22, 161]
LAVI, mL/m^2^	39 [29, 54]	45 [35, 55]	38 [28, 53]
LVDd, mm	47 [43, 52]	44 [41, 48]	47 [43, 52]
LVDs, mm	30 [26, 36]	28 [25, 30]	30 [26, 36]
LVEF, %	59 [50, 63]	62 [58, 64]	59 [49, 63]
E/A	0.80 [0.60, 1.10]	0.70 [0.58, 0.89]	0.80 [0.61, 1.10]
Septal e′, cm/s	5.0 [4.0, 6.4]	4.2 [3.6, 5.0]	5.1 [4.0, 6.5]
Lateral e′, cm/s	6.1 [4.7, 8.1]	4.8 [4.0, 6.5]	6.3 [4.8, 8.2]
E/e′	11.2 [8.8, 15.1]	14.7 [12.5, 19.1]	10.9 [8.7, 14.8]
AV Vmax, m/s	1.4 [1.1, 1.8]	3.4 [3.0, 4.0]	1.4 [1.1, 1.7]
AV mean PG, mmHg	4.2 [3.0, 7.0]	27.0 [21.0, 38.0]	4.0 [2.8, 6.0]
AV Area, cm^2^	2.0 [1.4, 2.5]	0.9 [0.8, 1.0]	2.1 [1.7, 2.6]
AVAI, cm^2^/m^2^	1.2 [0.9, 1.5]	0.6 [0.5, 0.7]	1.3 [1.1, 1.6]
Other valvular diseases			
Moderate or greater AR (%)	103/951 (10.8)	13/70 (18.6)	90/881 (10.2)
Moderate or greater MR (%)	154/950 (16.2)	4/70 (5.7)	150/880 (17.0)
Moderate or greater TR (%)	88/951 (9.3)	6/70 (8.6)	82/881 (9.3)

*Note:* Values are presented as median [interquartile range] or *n*/*N* (%). Denominators reflect the number of patients with non‐missing data for each variable. The number and percentage of missing data for each variable, stratified by AS severity, are provided in Supporting Information S1: Table 4. Patients were categorized according to transthoracic echocardiographic assessment of AS severity.

Abbreviations: AR, aortic regurgitation; AS, aortic stenosis; AV, aortic valve; AVAI, aortic valve area index; BMI, body mass index; BNP, B‐type natriuretic peptide; BP, blood pressure; E/A, ratio of early to late diastolic mitral inflow velocity; E/e′, ratio of early mitral inflow velocity to mitral annular early diastolic velocity; LAVI, left atrial volume index; LVDd, left ventricular end‐diastolic diameter; LVDs, left ventricular end‐systolic diameter; LVEF, left ventricular ejection fraction; mPG, mean pressure gradient; MR, mitral regurgitation; TR, tricuspid regurgitation; Vmax, maximum transvalvular velocity.

The diagnostic performance of the eAS model for detecting moderate‐or‐greater AS in the external validation cohort is shown in Figure 3. The AUROC was 0.9632 (95% CI: 0.9471–0.9774). When applying the binarization threshold predefined in the development cohort, the model demonstrated a sensitivity of 92.9% (95% CI: 86.4–98.4) and a specificity of 87.9% (95% CI: 85.5–90.0). At the prevalence observed in this cohort, the PPV was 37.8% (95% CI: 30.4–45.2), the NPV was 99.4% (95% CI: 98.7–99.9), the LR+ was 7.65 (95% CI: 6.36–9.40), and the LR− was 0.08 (95% CI: 0.02–0.15). Comparable diagnostic performance was preserved after excluding patients with prior aortic valve replacement or transcatheter aortic valve implantation. In this subgroup, the eAS model achieved an AUROC of 0.9628 (95% CI: 0.9450–0.9767), with sensitivity and specificity remaining essentially unchanged (Figure 3B). In post hoc ablation analyses, PCG alone accounted for most of the model's discriminative performance, while the eAS model output provided substantial incremental diagnostic value beyond clinical variables and standard 12‐lead ECG intervals (Supporting Information Methods and Results, Supporting Information S1: Table 6 and 7). Additionally, the interval between PCG/ECG acquisition and echocardiography was 0 days for the majority of patients, and model performance remained consistent across sensitivity analyses restricted to shorter intervals (Supporting Information Methods and Results; Supporting Information S1: Table 8).

**Figure 3:**
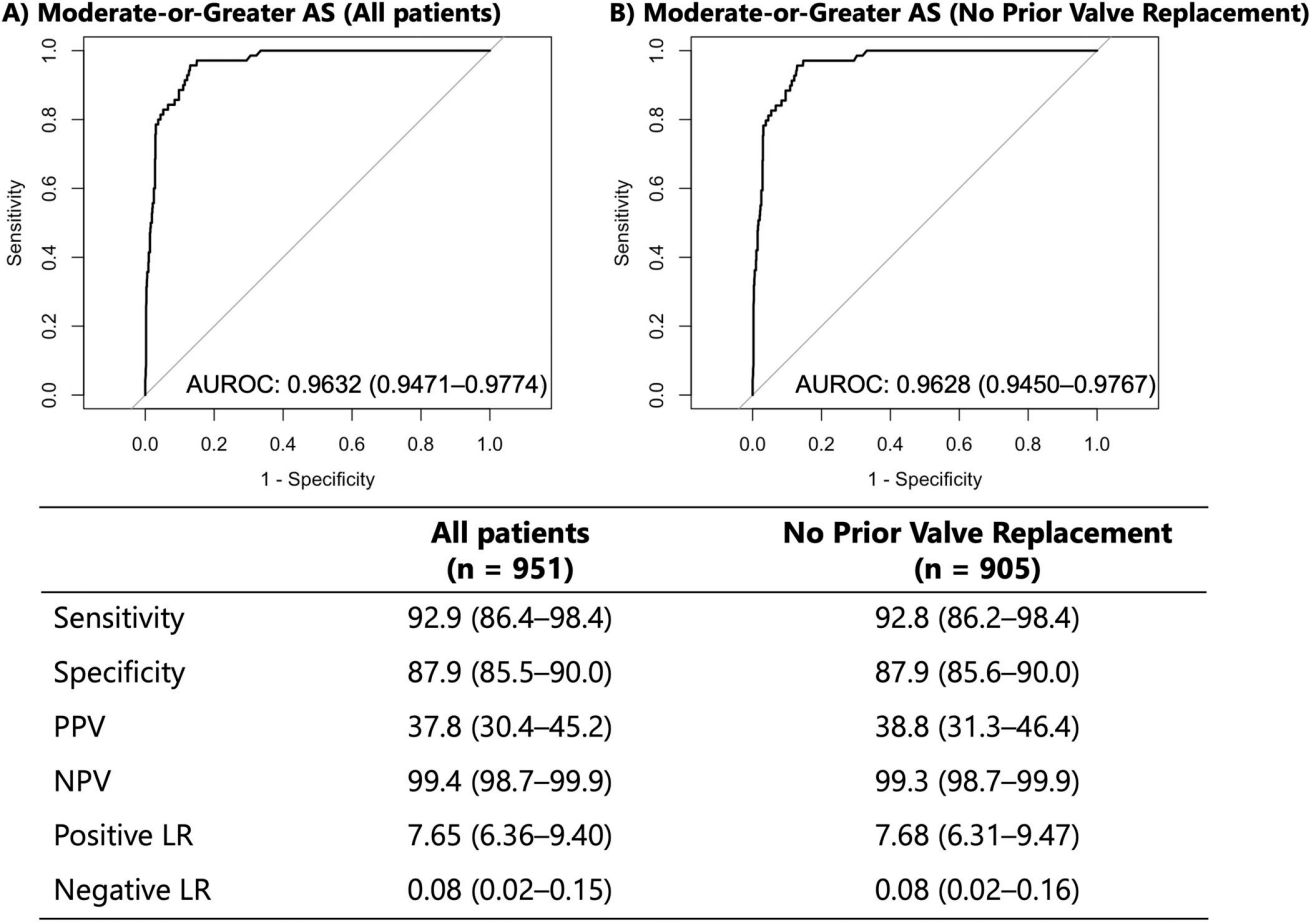
Performance of the eAS model for detecting moderate‐or‐greater AS. ROC curves and corresponding performance metrics of the eAS model for detecting moderate‐or‐greater AS in the external validation cohort. (A) Model performance in all patients (*n* = 951). (B) Model performance in patients without prior valve replacement (*n* = 905). The AUROC with 95% CIs is shown in each panel. Sensitivity, specificity, PPV, NPV, positive LR, and negative LR are summarized below the ROC curves. AS, aortic stenosis; AUROC, area under the receiver operating characteristic curve; CI, confidence interval; LR, likelihood ratio; NPV, negative predictive value; PPV, positive predictive value; ROC, receiver operating characteristic.

We subsequently evaluated the association between the continuous eAS model probability and representative echocardiographic parameters of AS severity as a complete case analysis for each variable. Patients classified as positive by the eAS model had higher AV Vmax and mean PG, and smaller AVA compared with those classified as negative (Figure 4A; Vmax, 2.6 [1.8–3.4] vs. 1.3 [1.1–1.6] m/s, *n* = 781; mean PG, 15.3 [7.0–26.9] vs. 3.8 [2.7–5.4] mmHg, *n* = 770; AVA, 1.12 [0.89–1.70] vs. 2.18 [1.75–2.62] cm^2^, *n* = 500).

**Figure 4:**
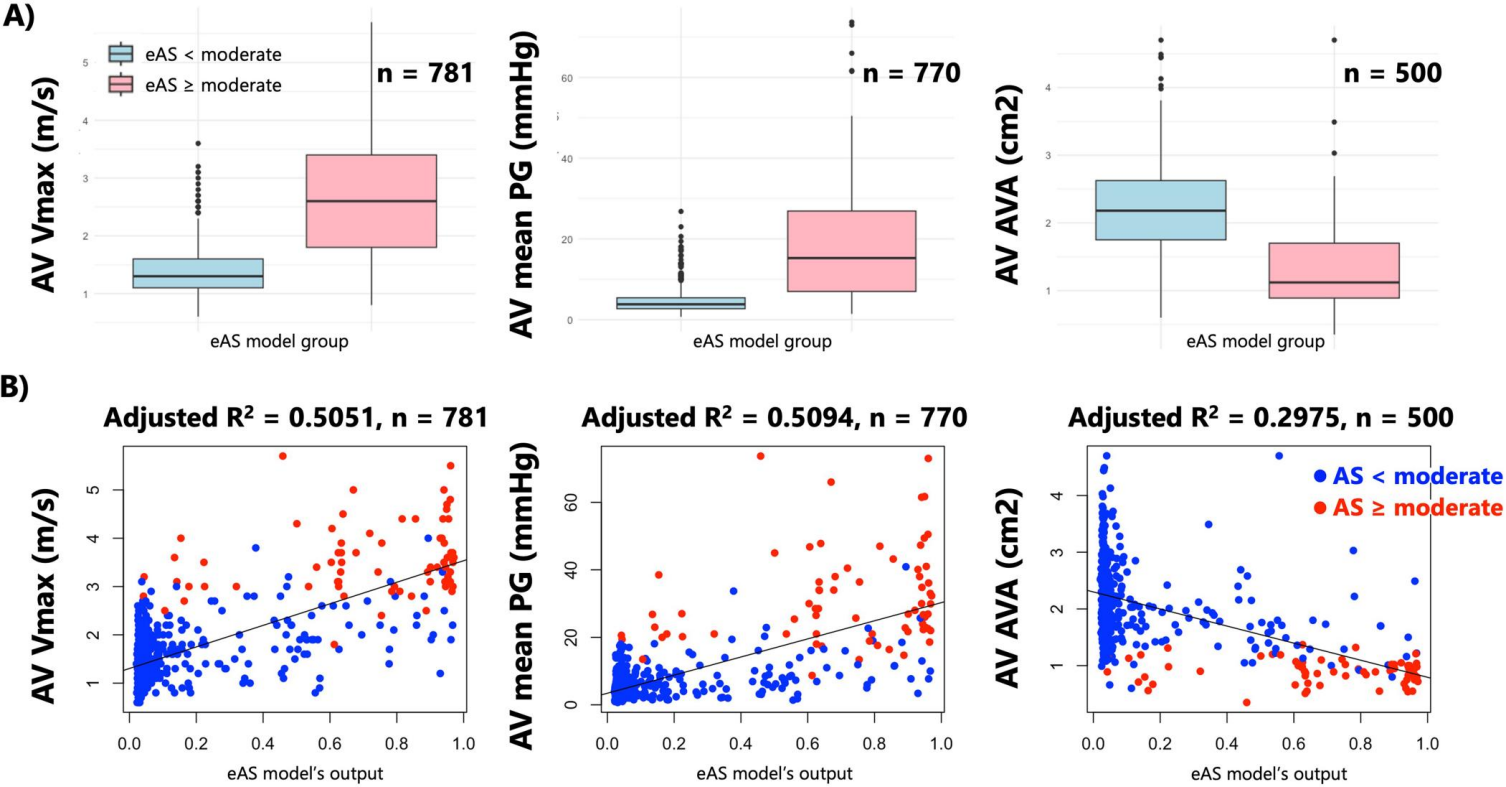
Association between the eAS model output and key echocardiographic indices of AS severity. Sample sizes differ across panels because of variable‐specific missingness in the underlying echocardiographic measurements (Supporting Information S1: Table 4). (A) Distribution of echocardiographic parameters—peak aortic valve velocity (AV Vmax), mean transvalvular pressure gradient (AV mean PG), and AVA—according to the eAS model–defined groups. (B) Scatter plots showing the association between the eAS model output and corresponding echocardiographic measures of AS severity. Adjusted *R* ^2^ values are shown for each analysis. Data points are color‐coded according to echocardiographic AS severity. AS, aortic stenosis; AV, aortic valve; AVA, aortic valve area; PG, pressure gradient.

When analyzed as a continuous variable, the model probability showed a strong association with AV Vmax (adjusted *R* ^2^ = 0.5051), mean PG (adjusted *R* ^2^ = 0.5094), and AVA (adjusted *R* ^2^ = 0.2975) (Figure 4B). These findings indicate that the eAS model output reflects the echocardiographic severity of AS in a continuous and biologically plausible manner.

Exploratory subgroup analyses were performed according to age, sex, BMI, cardiac rhythm, concomitant valvular heart disease, and LVEF in the external validation cohort. AUROC, sensitivity, and specificity estimates across subgroups are shown in Figure 5, with wide CIs in several subgroups. Sensitivity was below 80% in patients with moderate‐or‐greater MR, which included four patients with moderate‐or‐greater AS, while specificity was below 80% in patients with moderate‐or‐greater AR or TR. In an additional exploratory analysis according to AS hemodynamic phenotype, 25 of 26 patients with severe AS were correctly identified (Supporting Information S1: Table 9).

**Figure 5:**
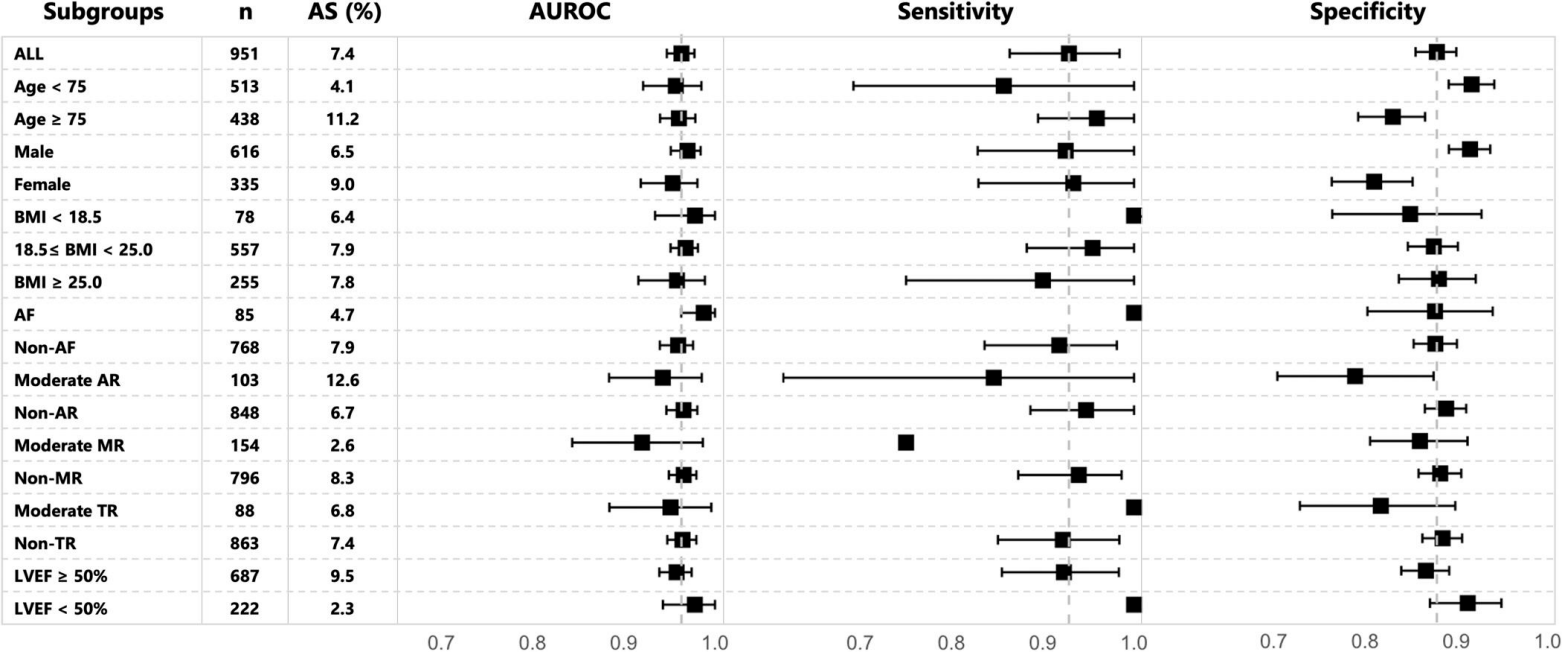
Subgroup analysis of the eAS model performance in the external validation cohort. Forest plots summarizing the performance of the eAS model across predefined clinical subgroups in the external validation cohort. For each subgroup, the AUROC, sensitivity, and specificity for detecting moderate‐or‐greater AS are shown with corresponding 95% CIs. The number of patients and the prevalence of moderate‐or‐greater AS are shown for each subgroup. Subgroups were defined according to age, sex, body mass index, AF, concomitant valvular heart disease, and LVEF. Missing data affected subgroup counts for BMI, AF, Moderate MR, and LVEF (Supporting Information S1: Table 4). AF, atrial fibrillation; AR, aortic regurgitation; AS, aortic stenosis; AUROC, area under the receiver operating characteristic curve; BMI, body mass index; LVEF, left ventricular ejection fraction; MR, mitral regurgitation; TR, tricuspid regurgitation.

Because AS severity was adjudicated from single‐reader echocardiographic reports without formal inter‐reader review, we performed a series of sensitivity analyses to characterize the influence of reference‐standard uncertainty on model performance, including stratification by AS severity (moderate or severe), evaluation using single echocardiographic criteria, restriction to cases with concordant Vmax/mean PG/AVA classification, focused analysis of AVA‐based discordant cases, and simulation‐based perturbation of echocardiographic measurements based on published inter‐observer variability. Across all these analyses, the diagnostic performance of the eAS model remained essentially unchanged, and apparent misclassifications were concentrated in borderline or discordant cases rather than reflecting a systematic reduction in model accuracy (Supporting Information Methods and Results; Supporting Information S1: Tables 10–13).

Although the raw model probabilities showed calibration drift in the external cohort, calibration was substantially improved after recalibration fit on the development cohort alone. The model also showed high average precision (0.692, 95% CI 0.568–0.801) and favorable net benefit across a range of threshold probabilities in decision curve analysis (Supporting Information Methods and Results; Supporting Information S1: Figures 1–3; Supporting Information S1: Tables 14–16).

As an error analysis, we conducted a detailed review of false‐negative (FN) cases (Supporting Information S1: Table 17). Among the five FN cases in the external validation cohort, three were located at the borderline between mild and moderate AS, with key echocardiographic parameters close to the diagnostic cutoffs. The remaining two cases exhibited multiple valvular regurgitations with complex hemodynamics. Although these two cases demonstrated faint systolic murmurs, they lacked the characteristic diminished second heart sound (S2) typically observed in AS patients.

Compared with true negatives, false positives were older, more frequently female, and had higher BNP and E/e′, with AS‐related echocardiographic indices (Vmax, mean PG, AVA, AVAI) closer to the moderate‐AS threshold; 21.5% had a pre‐existing diagnosis of mild AS versus 2.6% of true negatives (Supporting Information Results, Supporting Information S1: Table 18).

The representative PCG wavelet time–frequency representations for each category of the confusion matrix are shown in Supporting Information S1: Figure 4. Model‐derived attention maps indicated that the model frequently focused on acoustic activity during mid systole, as well as around the S2 and diastolic phases. These visual patterns were observed across both correctly and incorrectly classified cases, providing qualitative insight into the temporal components of PCG signals that contributed to model predictions.

## 4 Discussion

In this study, we developed a deep learning–based model that detects clinically significant AS (moderate‐or‐greater AS) using short‐duration (8‐s) PCG and single‐lead ECG recordings, and evaluated its performance in an independent, multicenter external validation cohort. The eAS model achieved excellent discriminative performance, with an AUROC of 0.9632. At the pre‐specified threshold, determined using the development cohort only, both sensitivity and specificity were high in the external validation cohort, and the LR (+LR 7.65 and −LR 0.08) demonstrated that the model meaningfully alters the diagnostic probability of AS. Importantly, the reference standard in the external validation cohort—echocardiographic findings—was determined at each site independently of the AI model's output, based on the TTE report generated as part of routine clinical care, with clinicians blinded to the Cardio‐EGG recordings and eAS model outputs; this design structurally minimizes the potential for sponsor bias. A limitation of this reference‐standard approach is the inherent measurement variability of echocardiography; however, the detailed sub‐analyses provided in Supporting Information S1: Tables 10–13 help address this limitation.

Because failing to identify a patient with clinically significant AS carries greater clinical consequences than performing an unnecessary echocardiogram, the pre‐specified Youden index threshold, which favors sensitivity, was considered clinically appropriate. A higher‐sensitivity threshold could be adopted if further minimizing missed cases is prioritized, at the cost of reduced specificity and PPV (Supporting Information S1: Table 15).

One possible explanation for the high diagnostic performance of the eAS model is that it successfully captured underlying disease‐related acoustic features of AS, rather than relying solely on superficial acoustic patterns. The model‐derived probability showed strong associations with key echocardiographic parameters, including Vmax, mean PG, and AVA, supporting the biological plausibility of the model output in relation to AS severity. This suggests that the model may have detected hemodynamic signatures—such as flow turbulence, pressure gradient–related vibrations, and temporal changes in systolic signals—that directly reflect the severity of AS.

Notably, comparable diagnostic performance was also preserved in patients without prior aortic valve replacement or transcatheter intervention, indicating that the model performance was not driven by post‐interventional valve characteristics but instead reflects intrinsic disease‐related features. Taken together, these findings highlight a key strength of the eAS model: its ability to provide clinically meaningful outputs from short‐duration PCG and ECG recordings in a fully independent external cohort.

On the other hand, the subgroup analyses revealed apparent variations in model performance. Although not statistically conclusive, patients with AF or moderate‐or‐greater TR tended to show higher sensitivity, whereas those with moderate‐or‐greater MR appeared to show reduced sensitivity. However, the apparent reduction in sensitivity observed in the MR subgroup should be interpreted in the context of the extremely small number of AS‐positive cases. Specifically, only four patients with concomitant moderate‐or‐greater MR had moderate‐or‐greater AS, and a single misclassification resulted in an apparent sensitivity of 75%. Rather than sensitivity, a more clinically relevant observation is that the eAS model maintained high specificity (86%) despite the presence of moderate‐or‐greater MR, correctly identifying patients without clinically significant AS. This finding indicates that the model is capable of appropriately discriminating non‐AS cases even in the presence of prominent regurgitant murmurs, representing a notable strength supporting its potential role as a diagnostic aid in routine cardiology practice. In contrast, concomitant MR can substantially reduce forward stroke volume across the aortic valve, leading to lower transvalvular velocities and overlapping systolic murmurs that may obscure AS‐related acoustic signatures. Such hemodynamic interactions are also recognized limitations in echocardiographic assessment and may partly explain the subgroup‐specific performance patterns observed in this study.

Furthermore, CNN‐based Grad‐CAM visualization provided additional interpretability to the model outputs, complementing the quantitative diagnostic performance metrics. The attention maps indicated that the eAS model focused not only on late systolic acoustic features but also on signals around the second heart sound and diastolic phases. These temporal regions often require expert interpretation during conventional auscultation and are consistent with established pathophysiological mechanisms of moderate‐or‐greater AS. The observed attention patterns suggest that the model integrates multiple components of the cardiac cycle, reflecting the complexity inherent in AS assessment.

Previous studies have shown that murmur intensity correlates with Vmax, but its diagnostic value is limited by body habitus [28]. In contrast, the eAS model demonstrated stable diagnostic performance across a wide range of patient characteristics. Moreover, although prior studies have explored heart sound‐based AS detection [23, 24, 29], few have validated their models in a fully independent, multicenter external cohort. By design, the present study addressed this gap by evaluating the eAS model in a real‐world, multicenter outpatient population. AI has also been applied to other stages of the AS management pathway, such as fully automated CT‐based measurement of aortic valve and root anatomy to assist procedural planning for TAVR or SAVR [30]. In contrast, the present approach targets an earlier step in the diagnostic pathway, using a brief, low‐cost acoustic and electrical signal to support the decision to refer patients from auscultation to echocardiography.

## 5 Limitations

This study has several limitations. First, the statistical design was powered only for the primary outcome, whereas subgroup analyses were exploratory. Confirmation of subgroup findings will require validation in larger cohorts, particularly for less prevalent clinical and hemodynamic subgroups.

Second, the study population consisted of patients referred for echocardiography, and the resulting selection and spectrum bias preclude extrapolation of these findings to a true asymptomatic screening population. As shown in Supporting Information S1: Table 16, the PPV varies substantially with disease prevalence, and this should be carefully considered when applying the model in settings with a different pretest probability of AS, such as population‐based screening.

Third, the definition of reference labels in borderline cases, particularly mild‐to‐moderate AS, remains inherently challenging, consistent with previously reported discordant grading among echocardiographic criteria in moderate AS [31]. Sensitivity analyses using alternative reference‐standard definitions—including single‐criterion thresholds, concordant‐case restriction, and simulated measurement —indicated that model performance was not substantially affected by this uncertainty (Supporting Information Methods and Results; Supporting Information S1: Tables 10–13); nonetheless, because AS severity was determined by single‐site, non‐centrally adjudicated interpretation without formal inter‐observer assessment, some residual uncertainty cannot be excluded.

Fourth, the study population consisted predominantly of Asian (Japanese) patients. Although no clear evidence currently suggests systematic ethnic differences in heart sound characteristics, the generalizability of the eAS model to other ethnic populations warrants further investigation.

Finally, the eAS model was not designed to detect other clinically significant valvular diseases, including MR, AR and TR. Given that concomitant MR and AR might worsen the prognosis of patients with AS [32, 33], and that an effective diagnostic‐support tool should ideally detect multiple clinically significant valvular heart diseases, further algorithm development is warranted.

## 6 Conclusion

In this multicenter study, we externally validated a deep learning–based model (eAS model) using PCG and single‐lead ECG signals to detect moderate‐or‐greater AS. The model demonstrated high diagnostic performance, and its output scores were associated with key echocardiographic measures of AS severity. These findings suggest that AI‐based PCG/ECG analysis may support the referral decision from auscultation to echocardiography within the diagnostic pathway for AS. Further studies are warranted to validate the model in broader populations and to extend the approach to the detection of other clinically significant valvular heart diseases.

## Author Contributions

Conceptualization: Shumpei Saito, Masanobu Ishii, Shimpei Ogawa, Mitsuru Ohishi, Kenichi Tsujita. Methodology: Shumpei Saito, Masanobu Ishii, Shimpei Ogawa, Hiroshi Seki, Koshiro Ikeda, Youhei Kayano, Yuhei Yasui, Ginga Sato. AI model development: Hiroshi Seki, Koshiro Ikeda. Data acquisition: Mitsuru Ohishi, Takuro Kubozono, Naritatsu Saito, Eri Toda Kato, Masahiro Yamada, Shunsuke Natori, Yuki Kunikane, Takafumi Yokomatsu, Masashi Kato, Yasuaki Sagara, Nami Uchiyama, Nobuhiko Atsuchi, Shota Kawahara, Shoji Natsugoe, Noriaki Tabata, Kenichi Tsujita. Echocardiographic diagnosis and adjudication: Mitsuru Ohishi, Takuro Kubozono, Naritatsu Saito, Eri Toda Kato, Masahiro Yamada, Shunsuke Natori, Yuki Kunikane, Takafumi Yokomatsu, Masashi Kato, Nami Uchiyama, Nobuhiko Atsuchi, Shoji Natsugoe, Noriaki Tabata, Kenichi Tsujita. Statistical analysis: Shumpei Saito, Masanobu Ishii, Hiroshi Seki, Koshiro Ikeda, Yuhei Yasui, Kenichi Tsujita. Interpretation of data: all authors. Drafting of manuscript: Shumpei Saito. Critical revision: all authors.

## Ethics Statement

This study was conducted in accordance with the Declaration of Helsinki and was approved by the Institutional Review Boards of Kumamoto University Hospital (Approval No. 2030), Saiseikai Kumamoto Hospital (Approval No. 932), Hokkaido Social Work Association Furano Hospital (Approval No. 21‐10), and Kajiki Onsen Hospital (Approval No. 507). Written informed consent was obtained from all participants prior to enrollment.

## Conflicts of Interest

Kenichi Tsujita, Mitsuru Ohishi, Naritatsu Saito, Masahiro Yamada, Shunsuke Natori, Takafumi Yokomatsu, Yasuaki Sagara, Nobuhiko Atsuchi and Shoji Natsugoe received joint research funding or commissioned research funding from AMI Inc. Shumpei Saito, Hiroshi Seki, Koshiro Ikeda, Youhei Kayano, and Ginga Sato are employees of AMI Inc. Shimpei Ogawa and Yuhei Yasui are board members of AMI Inc.

## Supporting information


Supporting File

## Data Availability

The deidentified participant data are not publicly available due to ethical and legal restrictions but may be available from the corresponding author upon reasonable request and with appropriate institutional approval.
