# Supplementary material for: Diagnostic Performance of a Deep Learning Model Using Phonocardiogram and Single‐Lead ECG Signals for Detecting Moderate‐or‐Greater Aortic Stenosis: Multicenter External Validation
Source: Clin Cardiol. 2026 Oct 15;49(10):e70498. doi: 10.1002/clc.70498 (PMC13670203; doi:10.1002/clc.70498)
Supplement: Supplementary file 1 [file CLC-49-e70498-s001.docx]

**Supplemental material**

**Title:**

Diagnostic Performance of a Deep Learning Model Using Phonocardiogram and Single-Lead ECG Signals for Detecting Moderate-or-Greater Aortic Stenosis: Multicenter External Validation

Table of Contents

[Supplemental Methods	3](#_Toc241064617)

[Model development	3](#_Toc241064618)

[**Measurement Sites, Sampling Rates, and Preprocessing of PCG/ECG Signals**	3](#_Toc241064619)

[**Handling of Class Imbalance and Misclassified Cases**	3](#_Toc241064620)

[**Architecture and Training Conditions of the 1D-CNN Model**	4](#_Toc241064621)

[**Architecture and Training Conditions of the wav2vec-Based Model**	4](#_Toc241064622)

[Independence of External validation cohort from model development	5](#_Toc241064623)

[**Self-Supervised Pre-training Dataset**	5](#_Toc241064624)

[**Pre-specification of Model and Threshold, and Transparency of the External Validation Analysis**	6](#_Toc241064625)

[Statistical analysis	8](#_Toc241064626)

[**Modality-Specific Ablation Analysis**	8](#_Toc241064627)

[**Incremental Value of the eAS Model Beyond Clinical and Electrocardiographic Variables**	8](#_Toc241064628)

[**Sensitivity Analysis by PCG/ECG–Echocardiography Interval**	9](#_Toc241064629)

[**Hemodynamic classification of aortic stenosis**	9](#_Toc241064630)

[**Reference-Standard Uncertainty in Aortic Stenosis Severity Classification: Sensitivity Analyses**	9](#_Toc241064631)

[**Calibration Analysis**	11](#_Toc241064632)

[**Precision-Recall Analysis**	12](#_Toc241064633)

[**Decision Curve Analysis**	12](#_Toc241064634)

[**Alternative Classification Thresholds**	12](#_Toc241064635)

[**Projected Diagnostic Yield Across Assumed Prevalence Scenarios**	12](#_Toc241064636)

[Supplemental Results	14](#_Toc241064637)

[Supplemental Table 4. Number and Percentage of Missing Data by Variables and AS severity.	14](#_Toc241064638)

[Reference Standard Missingness	16](#_Toc241064639)

[Modality-Specific Ablation Analysis	17](#_Toc241064640)

[Incremental Value of the eAS Model Beyond Clinical and Electrocardiographic Variables	18](#_Toc241064641)

[Sensitivity Analysis by PCG/ECG–Echocardiography Interval	19](#_Toc241064642)

[Hemodynamic classification of aortic stenosis	20](#_Toc241064643)

[Reference-Standard Uncertainty in Aortic Stenosis Severity Classification: Sensitivity Analyses	21](#_Toc241064644)

[Calibration Analysis	25](#_Toc241064645)

[Precision-Recall Analysis	27](#_Toc241064646)

[Decision Curve Analysis	28](#_Toc241064647)

[Alternative Classification Thresholds	29](#_Toc241064648)

[Projected Diagnostic Yield Across Assumed Prevalence Scenarios	30](#_Toc241064649)

[False Negative Case Analysis	31](#_Toc241064650)

[Characterization of False-Positive Cases	33](#_Toc241064651)

[Qualitative Interpretability Analysis	36](#_Toc241064652)

[Supplemental References	38](#_Toc241064653)

# **Supplemental Methods**

## **Model development**

### **Measurement Sites, Sampling Rates, and Preprocessing of PCG/ECG Signals**

PCG/ECG recordings obtained at the second right sternal border (2RSB), second left sternal border (2LSB), fourth left sternal border (4LSB), and fifth left midclavicular line (5LMCL) were used for model development. As described in the main text, recordings obtained at the 4LSB were used for the primary performance evaluation in the external validation cohort.

ECG was acquired simultaneously with PCG as a single-lead bipolar ECG using electrodes embedded in the Cardio-EGG device. At each measurement site, the device was positioned so that the negative electrode (–) was oriented cranially and the positive electrode (+) caudally. The native sampling rates of PCG and ECG were 8,000 Hz and 500 Hz, respectively.

PCG signals were normalized to zero mean and unit variance. For preprocessing of the 1D-CNN model, a 900-Hz low-pass filter was applied to the PCG signal. For preprocessing of the wav2vec-based model, both PCG and ECG signals were resampled to 2,000 Hz, and a 0.05–200 Hz band-pass filter was applied to the ECG signal. No denoising or signal-enhancement processing was applied.

### **Handling of Class Imbalance and Misclassified Cases**

The distribution of aortic stenosis (AS) severity in the training dataset was imbalanced. To mitigate this, a weighted loss function based on the ratio of positive to negative cases at each AS severity cutoff was used.

After the first stage of 1D-CNN model training, inference was performed on the development data to identify misclassified cases. Because these cases could include annotation errors or cases that were inherently difficult to classify, the loss weight assigned to these cases was reduced in subsequent training to limit their influence on model parameter updates. The self-attention and fully connected layers were then randomly reinitialized before the second stage of training. The case-specific loss weights used in the second stage of 1D-CNN training were also applied to supervised training of the wav2vec-based model.

### **Architecture and Training Conditions of the 1D-CNN Model**

The 1D-CNN model consisted of a deep convolutional neural network, a self-attention pooling layer, and six fully connected layers. The deep convolutional neural network comprised a stem convolutional layer followed by 10 CNN blocks, each containing a convolutional layer, a squeeze-and-excitation (SE) module, and a pointwise convolution.

AdamW was used as the optimizer, and binary cross-entropy was used as the loss function. Using the case-specific loss weights described above, the second stage of training was performed after randomly reinitializing the self-attention and fully connected layers.

During training, data augmentation was applied, including Gaussian noise, background noise, speed perturbation, low-pass filtering, and high-pass filtering. Background noise consisted of speech, environmental sounds, and music.

### **Architecture and Training Conditions of the wav2vec-Based Model**

The feature encoder of the wav2vec-based model consisted of six 1-dimensional convolutional layers. The Transformer component comprised 16 blocks, of which the lowest 5 blocks were used during supervised learning. The resulting feature representations were converted into fixed-length vectors by max pooling and then passed through four fully connected layers.

AdamW was used as the optimizer, and binary cross-entropy with label smoothing was used as the loss function. The same case-specific loss weights used in the second stage of 1D-CNN training were applied.

During training, data augmentation included Gaussian noise, background noise, speed perturbation, low-pass filtering, and high-pass filtering, in addition to time masking, baseline wander, and polarity inversion.

**Supplemental Table 1. Hyperparameters used in supervised learning**

|  | **CNN (1st stage)** | **CNN (2nd stage)** | **wav2vec** |
| --- | --- | --- | --- |
| batch size | 320 | 320 | 256 |
| epoch/iterations† | 250 | 45 | 5000† |
| learning rate | 1.00E-03 | 1.00E-04 | 1.00E-05 |
| optimizer | AdamW | AdamW | AdamW |
| loss | binary cross entropy | binary cross entropy | binary cross entropy |

## **Independence of External validation cohort from model development**

### **Self-Supervised Pre-training Dataset**

Self-supervised pre-training of the PCG/ECG model used data from 6 of the 10 sites comprising the Development cohort (Sites A–F in Supplemental Table 2). Eligible cases were those with available PCG/ECG waveform files at these 6 sites and complete severity labels for AS, aortic regurgitation (AR), and mitral regurgitation (MR). Recordings for which PCG/ECG waveform files were unavailable included those in which heart sounds were judged by the trained healthcare professional at the time of measurement to have been inadequately captured; these were excluded from analysis. This judgment, however, was based solely on the PCG/ECG waveforms and audio and was made independently of all clinical information, including age, sex, and the reference label.

Although AS severity labels were available in the database, they were not used as inputs, objectives, or loss terms, nor for optimization of model parameters, during self-supervised pre-training; feature representations were learned from the PCG/ECG waveforms alone.

For supervised model development, 5-fold cross-validation was used, with the Training/Development split performed at the patient level. When multiple PCG/ECG recordings were obtained from the same patient, all recordings from that patient — regardless of measurement site or recording conditions — were assigned to the same fold, so that no patient's data were split between the Training and Development folds. Because the 6 sites used for self-supervised pre-training were fully contained within the 10 sites used for supervised model development, some patients used for self-supervised pre-training were also included in the Development fold of supervised model development (although AS labels were not used during self-supervised pre-training). Performance evaluation on the Development fold is therefore not regarded as an internal validation fully independent of the model development process, including self-supervised pre-training.

In contrast, the External validation cohort comprised 3 sites entirely distinct from the 10 sites used for self-supervised pre-training and supervised model development (Supplemental Table 2), with no overlap in either patients or sites. PCG/ECG waveforms from the External validation cohort were not used for self-supervised pre-training, supervised training, model selection, determination of the ensemble ratio, determination of the classification threshold, or any other hyperparameter tuning. The primary evaluation of the model's generalizability in this study was based on this fully independent External validation cohort.

### **Pre-specification of Model and Threshold, and Transparency of the External Validation Analysis**

The final prediction score was calculated by ensembling the outputs of the 1D-CNN and wav2vec-based models, yielding a continuous value ranging from 0 to 1. The ensemble ratio was determined to maximize AUROC in the Development data, and the binary classification threshold of 0.148, used to identify moderate-or-greater AS, was determined based on the Youden index in the Development data. Both were fixed prior to analysis of the External validation cohort, and no changes were made based on the results of the External validation cohort.

The primary analysis in the External validation cohort — evaluation of discriminative performance by AUROC and evaluation of binary classification performance using the pre-fixed threshold — was specified before the final performance analysis was conducted. However, a formal statistical analysis plan (SAP) was not prepared, and this analysis is not characterized as a fully pre-specified analysis.

Prior to finalizing the model and threshold, investigators reviewed the External validation cohort dataset, including outcome labels, to confirm data quality and adequacy of the sample size for validation; therefore, the model developers cannot be considered to have been technically fully blinded to the External validation cohort labels. However, the classification threshold of 0.148 was a value derived through a pre-specified, rule-based procedure using only the Development cohort, and this review did not result in any change to the composition of the Development cohort or the calculated threshold value. No retraining of the model, changes to hyperparameters, changes to the ensemble ratio, or changes to the classification threshold were made based on the results of the External validation cohort.

**Supplemental Table 2. Data Sources and Timeline for Model Development and External Validation**

|  |  | **Site** | **Period** | **Number of Patients** |
| --- | --- | --- | --- | --- |
| **Training & Development Cohort** | |  |  | **1,589** |
|  | Self-supervised pre-training | 6 site (A~F) | Feb 2021~July 2023 | 1,384 |
|  | Supervised deep-learnning | 10 site (A~J) | Feb 2021 ~ July 2023 | 1,589 |
|  | Model/Threshold Locked |  | 07-Sep-24 |  |
| **External Validation Cohort** | |  |  | **951** |
|  | Data collection | 3 site (K~M) | Feb 2021~ Dec 2021 |  |
|  | Validation analysis initiated |  | 13-Sep-24 |  |

*No site, patient, or waveform overlap existed between the Training/Development cohort (Sites A–J) and the External Validation cohort (Sites K–M). The self-supervised pre-training dataset comprised a subset of the Training/Development cohort available under data-sharing agreements in place at the time of pre-training.*

## **Statistical analysis**

### **Modality-Specific Ablation Analysis**

To evaluate the relative contribution of each input modality to the diagnostic performance of the eAS model, single-modality (PCG-only and ECG-only) and combined-modality (PCG+ECG) models were trained using the wav2vec architecture alone. This analysis used a single representative architecture to probe modality-specific contribution and does not constitute a full ablation of the CNN–Transformer ensemble architecture used in the primary analysis. Each model's classification threshold was determined using the Youden index in the development cohort and applied, unchanged, to the external validation cohort.

### **Incremental Value of the eAS Model Beyond Clinical and Electrocardiographic Variables**

To assess whether the eAS model output provides diagnostic value beyond routinely available clinical and 12-lead electrocardiographic (ECG) information, nested logistic regression models were compared. Reduced models included clinical variables (age, sex, BMI, atrial fibrillation, hemodialysis status, and heart rate) with or without standard 12-lead ECG intervals (PR, QRS, and corrected QT interval, and the Sokolow–Lyon voltage index); full models additionally included the eAS model's predicted probability. Models were fit in the development cohort and evaluated in the external validation cohort using complete cases for each comparison. Model fit was compared using the likelihood ratio test (development cohort), and discrimination was compared using the change in AUROC (ΔAUROC) with 95% confidence intervals from 2,000 bootstrap resamples (external validation cohort). Variance inflation factors were checked to rule out multicollinearity (all <1.2).

### **Sensitivity Analysis by PCG/ECG–Echocardiography Interval**

As a sensitivity analysis, model performance was re-evaluated after restricting the external validation cohort to patients with a same-day PCG/ECG-echocardiography interval, and separately to those with an interval of ≤7 days and ≤30 days, to assess whether the time interval between PCG/ECG acquisition and echocardiography affected model performance.

### **Hemodynamic classification of aortic stenosis**

Patients with severe AS were further classified according to flow-gradient patterns. High-gradient severe AS was defined as a peak aortic jet velocity ≥4.0 m/s or mean transvalvular pressure gradient ≥40 mmHg. Among patients with low-gradient severe AS, low flow was defined as a stroke volume index ≤35 mL/m². Low-flow, low-gradient AS was further classified as classical (LVEF <50%) or paradoxical (LVEF ≥50%), reflecting well-recognaized diagnostic challenges in this population.^1^ Patients with a stroke volume index >35 mL/m² were classified as normal-flow, low-gradient severe AS. When stroke volume index was unavailable, patients were categorized as low-gradient severe AS with unknown flow status.

### **Reference-Standard Uncertainty in Aortic Stenosis Severity Classification: Sensitivity Analyses**

Because AS severity was adjudicated from single-site echocardiographic reports without formal central reading or multi-reader review, we performed four complementary sensitivity analyses to characterize the influence of reference-standard uncertainty on model performance. First, model performance was stratified by AS severity (moderate vs. severe) to assess whether diagnostic performance differed according to disease severity; because specificity is calculated from the same negative-case denominator (n=881) in both strata, only sensitivity was compared. Second, model performance was re-evaluated using each of the three echocardiographic criteria — peak aortic valve velocity (Vmax, cutoff=3.0 m/s), mean transvalvular pressure gradient (mean PG, cutoff=20 mmHg), and aortic valve area (AVA, cutoff=1.0 cm^2^) — individually as the reference standard, and separately restricted to cases in which all three criteria were concordant in classification direction (concordant cases). Third, because AVA alone showed lower discriminative performance than Vmax or mean PG alone, we further examined the 142 cases meeting the AVA≤1.5 cm² criterion, cross-tabulating the model's binary prediction against the final adjudicated diagnosis, to clarify the basis of AVA-related discordance. Fourth, to evaluate the impact of measurement variability inherent to echocardiographic assessment, we performed a Monte Carlo perturbation analysis. For each of Vmax, mean PG, and AVA, independent Gaussian perturbations were applied to the observed values, with standard deviations derived from previously reported inter-observer coefficients of variation (Vmax, 2.00%; mean PG, 8.53%; AVA, 7.67%) applied to the moderate/severe severity cutoffs (Vmax 3.0/4.0 m/s; mean PG 20/40 mmHg; AVA 1.5/1.0 cm²) (Supplemental Table 3). For each parameter, 2,000 Monte Carlo simulations were performed; reference labels were re-adjudicated using the perturbed values, and model performance was recalculated and summarized as the median and 95% simulation interval.

**Supplemental Table 3. Derivation of Perturbation Standard Deviations for Reference-Standard Echocardiographic Parameters**

| **Parameter** | **Inter-observer CV** | **Moderate AS Threshold** | **Severe AS Threshold** | **Moderate σ** | **Severe σ** |
| --- | --- | --- | --- | --- | --- |
| Vmax | 2.00% | 3.0 m/s | 4.0 m/s | 0.06 | 0.08 |
| Mean PG | 8.53% | 20 mmHg | 40 mmHg | 1.71 | 3.41 |
| AVA | 7.67% | 1.5 cm² | 1.0 cm² | 0.12 | 0.08 |

*The perturbation standard deviation (σ) for each parameter was calculated as σ = CV × μ, where μ represents the diagnostic threshold value (moderate or severe AS cutoff) used as the representative measurement. Inter-observer CVs were obtained from a previously published reproducibility study.*^2^ *All σ values are rounded to two decimal places*

*Abbreviations: CV, coefficient of variation; PG, pressure gradient; AVA, aortic valve area.*

### **Calibration Analysis**

Calibration of the eAS model's predicted probabilities was assessed using calibration plots, in which the LOWESS-smoothed observed probability of moderate-or-greater AS was plotted against the predicted probability, separately for the development cohort and the external validation cohort. Calibration was summarized using the calibration intercept and slope from a logistic recalibration model, together with the Brier score. Because the raw (uncalibrated) model probabilities showed calibration drift in the external validation cohort, a logistic recalibration model was subsequently fit using the development cohort only and applied, unchanged, to the external validation cohort; calibration was reassessed after recalibration using the same metrics. Pointwise 95% confidence intervals for the calibration curves were estimated using 2,000 bootstrap resamples.

### **Precision-Recall Analysis**

Precision-recall curves were constructed for the development and external validation cohorts, plotting precision (positive predictive value) against recall (sensitivity) across all classification thresholds. Average precision (AP), equivalent to the area under the precision-recall curve, was calculated as a threshold-independent summary measure. Pointwise 95% confidence intervals for the curve and for AP were estimated using 2,000 bootstrap resamples.

### **Decision Curve Analysis**

Decision curve analysis was performed using the recalibrated model probabilities in the external validation cohort to estimate the net benefit of using the eAS model to guide referral for echocardiography, across threshold probabilities ranging from 0.0 to 0.8. Net benefit was compared with the default strategies of testing all patients ("treat all") and testing no patients ("treat none").

### **Alternative Classification Thresholds**

In addition to the pre-specified Youden index threshold (0.148) used in the primary analysis, thresholds achieving a target sensitivity of 95% and 99% in the development cohort were identified. Each threshold was determined using the development cohort only and then applied, unchanged, to the external validation cohort, to illustrate the trade-off between sensitivity and specificity/PPV at alternative operating points.

### **Projected Diagnostic Yield Across Assumed Prevalence Scenarios**

To illustrate how diagnostic yield and predictive values vary with disease prevalence, the number of true-positive, true-negative, false-positive, and false-negative classifications per 1,000 individuals tested, along with PPV and NPV, were projected by applying the sensitivity and specificity of the eAS model at the Youden index threshold to the observed external cohort prevalence (7.4%) and three illustrative assumed prevalence scenarios (1%, 3%, 5%).

# **Supplemental Results**

## **Supplemental Table 4. Number and Percentage of Missing Data by Variables and AS severity.**

|  | Moderate-or-greater AS | | Less-than-moderate AS | |
| --- | --- | --- | --- | --- |
| n = 951 | NA Count | % | NA Count | % |
| Age, years | 0 | 0 | 0 | 0 |
| Sex, male (%) | 0 | 0 | 0 | 0 |
| BMI, kg/m^2^ | 1 | 1.43 | 60 | 6.81 |
| Systolic BP, mmHg | 11 | 15.71 | 153 | 17.37 |
| Atrial Fibrillation (%) | 5 | 7.14 | 93 | 10.56 |
| BNP, pg/mL | 8 | 11.43 | 141 | 16 |
| LAVI, mL/m^2^ | 8 | 11.43 | 194 | 22.02 |
| LVDd, mm | 0 | 0 | 0 | 0 |
| LVDs, mm | 0 | 0 | 1 | 0.11 |
| LVEF, % | 0 | 0 | 42 | 4.77 |
| E/A | 8 | 11.43 | 143 | 16.23 |
| Septal e′, cm/s | 4 | 5.71 | 58 | 6.58 |
| Lateral e′, cm/s | 20 | 28.57 | 278 | 31.56 |
| E/e′ | 4 | 5.71 | 68 | 7.72 |
| AV Vmax, m/s | 1 | 1.43 | 169 | 19.18 |
| AV mPG, mmHg | 0 | 0 | 181 | 20.54 |
| AV Area, cm^2^ | 0 | 0 | 451 | 51.19 |
| AVAI, cm2/m^2^ | 5 | 7.14 | 582 | 66.06 |
| Other Valvular diseases |  |  |  |  |
| Moderate or greater AR (%) | 0 | 0 | 0 | 0 |
| Moderate or greater MR (%) | 0 | 0 | 1 | 0.11 |
| Moderate or greater TR (%) | 0 | 0 | 0 | 0 |

*This table summarizes the number and percentage of missing values for each clinical and echocardiographic variable in the external validation cohort, stratified by aortic stenosis (AS) severity (moderate-or-greater AS vs. less-than-moderate AS). Percentages are calculated based on the total number of patients within each AS severity group.*

*Abbreviations: AS, aortic stenosis; BMI, body mass index; BP, blood pressure; BNP, B-type natriuretic peptide; LAVI, left atrial volume index; LVDd, left ventricular end-diastolic diameter; LVDs, left ventricular end-systolic diameter; LVEF, left ventricular ejection fraction; E/A, ratio of early to late diastolic mitral inflow velocity; e′, early diastolic mitral annular velocity; AV, aortic valve; Vmax, peak aortic valve velocity; PG, pressure gradient; AVA, aortic valve area; AVAI, aortic valve area index; AR, aortic regurgitation; MR, mitral regurgitation; TR, tricuspid regurgitation.*

## **Reference Standard Missingness**

Number of the three quantitative parameters used to define aortic stenosis (AS) severity—peak transvalvular velocity (Vmax), mean pressure gradient (mean PG), and aortic valve area (AVA)—that were recorded for each patient, stratified by AS severity classification. All patients classified as having mild-or-greater AS had at least two of the three parameters recorded. Among patients classified as having no AS, none of the three parameters were recorded in 167 of 838 patients (19.9%); in these cases, the absence of moderate-or-greater AS was based on the overall qualitative echocardiographic impression (e.g., normal leaflet motion without visible narrowing or increased transvalvular flow), consistent with routine clinical practice.

**Supplemental Table 5. Number of Quantitative Echocardiographic Parameters Available for AS Severity Classification in the External Validation Cohort**

| **AS severity** | **3 item available** | **2 item available** | **1 item available** | **0 item available** |
| --- | --- | --- | --- | --- |
| without AS | 384 | 274 | 13 | 167 |
| mild or more AS | 112 | 1 | 0 | 0 |

*Abbreviations: AS, aortic stenosis; Vmax, peak transvalvular velocity; PG, pressure gradient; AVA, aortic valve area.*

## **Modality-Specific Ablation Analysis**

Using the wav2vec architecture, the PCG-only model achieved an AUROC of 0.940, while the ECG-only model achieved an AUROC of 0.539, close to chance level. The combined PCG+ECG model achieved an AUROC of 0.965, numerically similar to and within the 95% confidence interval of the primary eAS model (CNN–Transformer ensemble; AUROC 0.963, 95% CI 0.947–0.977) (Supplemental Table 6).

**Supplemental Table 6. Modality-Specific Ablation Analysis Using a Single wav2vec-Based Architecture**

| **Model** | **Input modality** | **Architecture** | **External N** | **AUROC** | **AUPRC** | **Sensitivity** | **Specificity** | **PPV** | **NPV** |
| --- | --- | --- | --- | --- | --- | --- | --- | --- | --- |
| PCG-only model | PCG | wav2vec | 951 | 0.94 | 0.67 | 82.9% | 94.0% | 52.3% | 98.6% |
| ECG-only model | ECG | wav2vec | 951 | 0.539 | 0.084 | 11.4% | 90.8% | 9.0% | 92.8% |
| Combined model | PCG+ECG | wav2vec | 951 | 0.965 | 0.669 | 92.9% | 91.7% | 47.1% | 99.4% |
| Reference:  Primary eAS model | PCG+ECG | CNN-Transformer ensemble | 951 | 0.963  (0.947–0.977) | 0.692 (0.568–0.801) | 92.9% (86.4–98.4%) | 87.9% (85.5–90.0%) | 37.8% (30.4–45.2%) | 99.4% (98.7–99.9%) |

*To assess the relative contribution of PCG and ECG to the diagnostic performance of the eAS model, single-modality (PCG-only, ECG-only) and combined-modality models were trained using the wav2vec architecture alone, as a representative single-architecture probe of modality-specific contribution; this analysis does not constitute a full ablation of the CNN–Transformer ensemble architecture used in the primary analysis. Classification thresholds were determined using the Youden index in the development cohort and applied, unchanged, to the external validation cohort.*

*Abbreviations: AUROC, area under the receiver operating characteristic curve; AUPRC, area under the precision-recall curve; PPV, positive predictive value; NPV, negative predictive value.*

## **Incremental Value of the eAS Model Beyond Clinical and Electrocardiographic Variables**

Adding standard 12-lead ECG interval variables to the clinical model did not improve discrimination (ΔAUROC −0.002, 95% CI −0.031 to 0.029), despite a statistically significant improvement in model fit (likelihood ratio test, P<0.001). In contrast, adding the eAS model's predicted probability substantially improved discrimination, both beyond the clinical+ECG model (ΔAUROC 0.194, 95% CI 0.144–0.245) and beyond the clinical-only model (ΔAUROC 0.201, 95% CI 0.153–0.252) (Supplemental Table 7).

**Supplemental Table 7. Incremental Diagnostic Value of the eAS Model Beyond Clinical and ECG Variables**

| **Comparison** | **Added predictor block** | **N, Dev/Ext**  **(AS≥mod, %)** | **Baseline AUROC**  **(95% CI)** | **AUROC after adding predictor**  **(95% CI)** | **ΔAUROC**  **(95% CI)** | **LRT df,**  **P-value** |
| --- | --- | --- | --- | --- | --- | --- |
| Model A vs  Model B | 12-lead ECG intervals | 1075 (51, 4.7%)/  697 (58, 8.3)% | 0.741 (0.678–0.798) | 0.740 (0.678–0.794) | −0.002 (−0.031 to 0.029) | 4, <0.001 |
| Model B vs  Model C | eAS model output | 1075 (51, 4.7%)/  697 (58, 8.3)% | 0.740 (0.678–0.794) | 0.934 (0.893–0.966) | 0.194 (0.144–0.245) | 1, <0.001 |
| Model A vs  Model D | eAS model output | 1368 (64, 4.7%)/  871 (69, 7.9%) | 0.733 (0.674–0.786) | 0.934 (0.895–0.966) | 0.201 (0.153–0.252) | 1, <0.001 |

*This table presents nested logistic regression model comparisons assessing the incremental diagnostic value of 12-lead ECG intervals and the eAS model's predicted probability beyond clinical variables.*

*- Model A included clinical variables only (age, sex, BMI, atrial fibrillation, hemodialysis status, and heart rate).*

*- Model B added 12-lead ECG intervals (PR, QRS, and corrected QT interval, and the Sokolow–Lyon voltage index) to Model A.*

*- Model C added the eAS model's predicted probability to Model B.*

*- Model D added the eAS model's predicted probability directly to Model A, without ECG intervals.*

*Each row compares the two named models after adding the corresponding predictor block; models were fit in the development cohort and evaluated in the external validation cohort using complete cases for each comparison. ΔAUROC and 95% confidence intervals were estimated using 2,000 bootstrap resamples; P values are from the likelihood ratio test comparing model fit in the development cohort and do not necessarily correspond to a significant change in discrimination (see Model A vs Model B).*

*Abbreviations: AS, aortic stenosis; AUROC, area under the receiver operating characteristic curve; CI, confidence interval; df, degrees of freedom; LRT, likelihood ratio test.*

## **Sensitivity Analysis by PCG/ECG–Echocardiography Interval**

Among the 951 patients in the external validation cohort, the median interval between Cardio-EGG (PCG/ECG) acquisition and echocardiography was 0 days (mean 9.6 days, SD 17.2; IQR 0–13 days; range 0–74 days). In all cases, echocardiography was performed on the same day as, or after, Cardio-EGG acquisition (i.e., no patient underwent echocardiography before Cardio-EGG acquisition).

The interval between PCG/ECG acquisition and echocardiography was 0 days for the majority of patients (median 0 days; mean 9.6 days; IQR 0-13; range 0-74), and AUROC remained consistent across same-day (n=575, AUROC 0.962), ≤7-day (n=658, AUROC 0.966), ≤30-day (n=839, AUROC 0.959), and full-cohort (n=951, AUROC 0.963) analyses (Supplemental Table 8).

**Supplemental Table 8. Sensitivity Analysis of Model Performance Stratified by Interval Between PCG/ECG Acquisition and Echocardiography**

| Group | N | AUROC (95%CI) | Sensitivity (95%CI) | Specificity (95%CI) | PPV (95%CI) | NPV (95%CI) | TP | TN | FP | FN |
| --- | --- | --- | --- | --- | --- | --- | --- | --- | --- | --- |
| Same day (0 days) | 575 | 0.962 (0.938–0.979) | 0.927 (0.837–1.000) | 0.875 (0.845–0.900) | 0.362 (0.270–0.455) | 0.994 (0.985–1.000) | 38 | 467 | 67 | 3 |
| ≤7 days | 658 | 0.966 (0.945–0.981) | 0.936 (0.857–1.000) | 0.882 (0.858–0.906) | 0.379 (0.292–0.468) | 0.994 (0.987–1.000) | 44 | 539 | 72 | 3 |
| ≤30 days | 839 | 0.959 (0.939–0.975) | 0.918 (0.841–0.982) | 0.878 (0.854–0.900) | 0.371 (0.295–0.447) | 0.993 (0.986–0.999) | 56 | 683 | 95 | 5 |
| All patients  (0–74 days) | 951 | 0.963 (0.947–0.977) | 0.929 (0.864–0.984) | 0.879 (0.855–0.900) | 0.378 (0.304–0.452) | 0.994 (0.987–0.999) | 65 | 774 | 107 | 5 |
| Different days only (1–74 days) | 376 | 0.965 (0.937–0.987) | 0.931 (0.826–1.000) | 0.885 (0.852–0.917) | 0.403 (0.289–0.525) | 0.994 (0.984–1.000) | 27 | 307 | 40 | 2 |

*"All patients (0–74 days)" corresponds to the full external validation cohort and reproduces the primary analysis results reported in the main text. "Different days only" restricts the cohort to patients in whom echocardiography was performed on a different calendar day than PCG/ECG acquisition (interval 1–74 days), providing the most conservative test of whether a nonzero interval affects model performance.*

*Abbreviations: AUROC, area under the receiver operating characteristic curve; CI, confidence interval; PPV, positive predictive value; NPV, negative predictive value; TP, true positive; TN, true negative; FP, false positive; FN, false negative.*

## **Hemodynamic classification of aortic stenosis**

Among 70 patients with moderate or greater AS, 44 had moderate AS and 26 had severe AS. The distribution of AS phenotypes according to eAS model results is shown in Supplemental Table 9. The eAS model correctly identified 40 of 44 patients (90.9%) with moderate AS and 25 of 26 patients (96.2%) with severe AS.

**Supplemental Table 9. Distribution of Aortic Stenosis Phenotypes and eAS Model Classification**

| **Phenotype of Aortic Stenosis** | **N** | **FN** | **TP** |
| --- | --- | --- | --- |
| Moderate AS | 44 | 4 | 40 |
| High-gradient severe AS | 18 | 0 | 18 |
| NFLG severe AS | 5 | 0 | 5 |
| Paradoxical LFLG severe AS | 2 | 0 | 2 |
| Classical LFLG severe AS | – | – | – |
| LG severe AS, flow unknown | 1 | 1 | 0 |

*Severe AS was categorized according to flow-gradient phenotype as described in the Supplemental Methods. Flow status was unavailable in one patient. No patients had classical LFLG severe AS.*

*Abbreviations: AS, aortic stenosis; FN, false negative; TP, true positive; LFLG, low-flow, low-gradient; NFLG, normal-flow, low-gradient; LG, low-gradient; LVEF, left ventricular ejection fraction.*

## **Reference-Standard Uncertainty in Aortic Stenosis Severity Classification: Sensitivity Analyses**

Stratified by AS severity, sensitivity was 90.9% (95% CI 81.8–97.7; 40/44) for moderate AS and 96.2% (95% CI 88.5–100; 25/26) for severe AS; because specificity is calculated from the shared negative-case denominator, specificity was identical (87.9%) across strata (Supplemental Table 10).

**Supplemental Table 10. Sensitivity of the eAS Model Stratified by AS Severity**

| **AS severity** | **N** | **TP** | **FN** | **Sensitivity (95% CI)** |
| --- | --- | --- | --- | --- |
| Moderate | 44 | 40 | 4 | 90.9% (81.8–97.7%) |
| Severe | 26 | 25 | 1 | 96.2% (88.5–100%) |

*Abbreviations: AS, aortic stenosis; CI, confidence interval; FN, false negative; N, number of patients; TP, true positive.*

When each echocardiographic criterion was used individually as the reference standard, AVA alone showed a lower AUROC than Vmax or mean PG alone. Restricting the analysis to the 412 cases in which all three criteria were concordant (TP=58, FN=2, FP=36, TN=316; sensitivity 96.7%) yielded an AUROC and sensitivity that both exceeded the primary analysis (AUROC 0.963, sensitivity 92.9%), indicating that diagnostic difficulty was concentrated among discordant or borderline cases rather than reflecting a systematic limitation of the model (Supplemental Table 11).

**Supplemental Table 11. Diagnostic Performance of the eAS Model According to Different Echocardiographic Reference Criteria**

A) Classification counts

| **Reference standard** | **N** | **AS prevvalence** | **TP** | **FN** | **FP** | **TN** |
| --- | --- | --- | --- | --- | --- | --- |
| Vmax criterion (≥ 3.0 m/sec) | 781 | 8.5% | 61 | 5 | 92 | 623 |
| Mean PG criterion (≥ 20 mmHg) | 770 | 8.4% | 62 | 3 | 90 | 615 |
| AVA criterion (< 1.5 cm2) | 500 | 28.4% | 89 | 53 | 39 | 319 |
| Concordant Vmax/ Mean PG/AVA criteria | 412 | 14.6% | 58 | 2 | 36 | 316 |

B) Diagnostic performance measures

| **Reference standard** | **N** | **AUROC (95% CI)** | **Sensitivity (95% CI)** | **Specificity (95% CI)** | **PPV**  **(95% CI)** | **NPV**  **(95% CI)** | **pLR (95% CI)** | **nLR (95% CI)** |
| --- | --- | --- | --- | --- | --- | --- | --- | --- |
| Vmax criterion (≥ 3.0 m/sec) | 781 | 0.9565 (0.9351–0.9737) | 0.9242 (0.8485–0.9848) | 0.8713 (0.8448–0.8937) | 0.3987 (0.3506–0.4477) | 0.992 (0.9843–0.9984) | 7.18 (5.85–8.78) | 0.09 (0.02–0.17) |
| Mean PG criterion (≥ 20 mmHg) | 770 | 0.9625 (0.9447–0.9762) | 0.9538 (0.8923–  1) | 0.8723 (0.8468–0.8965) | 0.4079 (0.3631–0.4627) | 0.9951 (0.9889–  1) | 7.47 (6.18–9.34) | 0.05 (0–  0.12) |
| AVA criterion (< 1.5 cm2) | 500 | 0.8187 (0.7705–0.8644) | 0.6268 (0.5493–0.7042) | 0.8911 (0.8575–0.9218) | 0.6953 (0.6288–0.7686) | 0.8575 (0.8316–0.8842) | 5.75 (4.27–8.37) | 0.42 (0.33–0.51) |
| Concordant Vmax/ Mean PG/AVA criteria | 412 | 0.9784 (0.9649–0.9892) | 0.9667 (0.9167–  1) | 0.8977 (0.8636–0.929) | 0.617 (0.5463–0.6988) | 0.9937 (0.9841–  1) | 9.45 (7.06–13.61) | 0.04 (0–  0.09) |

*Abbreviations: AS, aortic stenosis; AUROC, area under the receiver operating characteristic curve; AVA, aortic valve area; CI, confidence interval; FN, false negative; FP, false positive; mean PG, mean transvalvular pressure gradient; N, number of patients; nLR, negative likelihood ratio; NPV, negative predictive value; pLR, positive likelihood ratio; PPV, positive predictive value; TN, true negative; TP, true positive; Vmax, peak aortic valve velocity.*

Among the 142 cases meeting the AVA≤1.5 cm² criterion, 70 were ultimately adjudicated as moderate-or-greater AS (all of which also met the AVA criterion), while 72 (50.7%) met the AVA criterion but were adjudicated as less-than-moderate AS based on integrated clinical judgment. Cross-tabulating model predictions against the adjudicated diagnosis in this subgroup showed that, of 53 cases apparently missed by the AVA-alone criterion, only 5 (9.4%) were true model false negatives — identical to the 5 false negatives identified in the primary analysis — while the remaining 48 (90.6%) were correctly classified as negative by the model, consistent with the adjudicated diagnosis. Similarly, of 89 cases classified as positive by the model, 24 corresponded to AVA-discordant cases adjudicated as less-than-moderate, accounting for 22.4% of the 107 false positives in the primary analysis (Supplemental Table 12).

**Supplemental Table 12. Cross-Tabulation of eAS Model Predictions and Adjudicated Final Diagnosis Among Patients Meeting the AVA-Based Reference Criterion (AVA ≤1.5 cm²)**

|  | **Adjudicated: Moderate-or-greater AS** | **Adjudicated: Less-than-Moderate AS** | **Total** |
| --- | --- | --- | --- |
| eAS model: positive | 65 | 24 | **89** |
| eAS model: negative | 5 | 48 | **53** |
| **Total** | **70** | **72** | **142** |

*Abbreviations: AS, aortic stenosis; AVA, aortic valve area.*

Across all three parameters and perturbation conditions, the absolute change in AUROC was small and label-flip rates remained low (Supplemental Table 13), indicating that, within the range of measurement variability modeled here, reference-standard measurement uncertainty did not substantially affect model performance.

**Supplemental Table 13. Sensitivity Analysis of Model Performance to Simulated Measurement Variability in Reference-Standard Echocardiographic Parameters**

| **Parameter** | **Severity Threshold** | **Perturbation SD (σ)** | **N** | **Pre-perturbation AUROC** | **Simulated**  **AUROC**  **(Med, 95% SI)** | **Label Change**  **Rate**  **(Med, 95% SI)** | **Negative-to-**  **Positive Change**  **(Med, 95% SI)** | **Positive-to-**  **Negative Changs**  **(Med, 95% SI)** |
| --- | --- | --- | --- | --- | --- | --- | --- | --- |
| Vmax | Moderate  (3.0 m/s) | 0.06 | 781 | 0.957 | 0.956  (0.952–0.962) | 0.8% (0.3–1.2%) | 0.7% (0.3–1.3%) | 0.0% (0.0–1.5%) |
| Vmax | Severe  (4.0 m/s) | 0.08 | 781 | 0.957 | 0.956  (0.951–0.964) | 0.8% (0.4–1.3%) | 0.8% (0.4–1.4%) | 0.0% (0.0–3.0%) |
| Mean PG | Moderate  (20 mmHg) | 1.71 | 770 | 0.963 | 0.963  (0.953–0.971) | 0.8% (0.3–1.4%) | 0.6% (0.1–1.0%) | 3.1% (0.0–9.2%) |
| Mean PG | Severe  (40 mmHg) | 3.41 | 770 | 0.963 | 0.956  (0.936–0.969) | 1.6% (0.9–2.3%) | 0.9% (0.4–1.4%) | 9.2% (3.1–15.4%) |
| AVA | Moderate  (1.5 cm²) | 0.12 | 500 | 0.819 | 0.817  (0.795–0.838) | 3.6% (2.2–5.0%) | 2.8% (1.7–4.5%) | 5.6% (2.1–8.5%) |
| AVA | Severe  (1.0 cm²) | 0.08 | 500 | 0.819 | 0.820  (0.802–0.839) | 2.4% (1.2–3.6%) | 2.2% (0.8–3.4%) | 3.5% (0.7–5.6%) |

*Model performance was recalculated after applying Gaussian perturbation to each echocardiographic parameter independently, based on published inter-observer coefficients of variation. Values are shown as median (95% simulation interval) across 2,000 Monte Carlo simulations per parameter.*

*Abbreviation: AS, aortic stenosis; AUROC, area under the receiver operating characteristic curve; AVA, aortic valve area; mean PG, mean transvalvular pressure gradient; SD, standard deviation; Vmax, peak aortic valve velocity.*

Taken together, these four analyses — severity-stratified sensitivity, single-criterion and concordant-case restriction, characterization of AVA-based discordance, and simulated measurement perturbation — indicate that the influence of reference-standard uncertainty on the reported model performance is limited.

## **Calibration Analysis**

Calibration of the raw (uncalibrated) model probabilities showed evidence of drift toward overestimation of AS risk in the external validation cohort (calibration slope 1.068, intercept −1.331; Brier score 0.045); after logistic recalibration fit on the development cohort alone, calibration improved substantially (slope 0.844, intercept 0.027; Brier score 0.038) (Supplemental Figure 1, Supplemental Table 14).

**Supplemental Figure 1. Calibration of the eAS Model in the Development and External Validation Cohorts**

*Calibration plots show the relationship between predicted probability (x-axis) and observed event probability (y-axis) for moderate-or-greater AS. From left to right: the development cohort with uncalibrated model probabilities; the external validation cohort with uncalibrated model probabilities, prior to recalibration; and the external validation cohort after logistic recalibration fit on the development cohort alone. The dashed diagonal line represents ideal calibration; the orange line represents a LOWESS-smoothed calibration curve; the shaded band represents the 95% bootstrap confidence interval (2,000 resamples). Corresponding calibration statistics are presented in Supplemental Table 14.*

**Supplemental Table 14. Calibration Performance of the eAS Model in the Development and External Validation Cohorts**

| **Metric** | **Development (Uncalibrated)** | **External (Uncalibrated)** | **External (Recalibrated)** |
| --- | --- | --- | --- |
| Calibration intercept | -1.609 (-2.004 to -1.265) | -1.331 (-1.698 to -0.990) | 0.027 (-0.396 to 0.508) |
| Calibration slope | 1.265 (1.112 to 1.503) | 1.068 (0.926 to 1.276) | 0.844 (0.732 to 1.009) |
| Brier score | 0.032 (0.027 to 0.038) | 0.045 (0.037 to 0.055) | 0.038 (0.028 to 0.047) |
| O:E ratio | 0.407 (0.335 to 0.486) | 0.534 (0.445 to 0.620) | 1.107 (0.927 to 1.295) |

*Calibration was assessed using LOWESS-smoothed calibration curves (Supplemental Figure 1) and summarized using the calibration intercept, calibration slope, Brier score, and observed-to-expected (O:E) event ratio, each estimated with 2,000 bootstrap resamples (95% CI). Recalibration was performed by fitting a logistic recalibration model on the development cohort only and applying it to the external validation cohort; the "External (Uncalibrated)" column was not used in fitting the recalibration model and reflects raw model probabilities prior to recalibration.*

*Abbreviations: CI, confidence interval; O:E, observed-to-expected.*

## **Precision-Recall Analysis**

The precision–recall curve showed an average precision (PR-AUC) of 0.728 (95% CI 0.631–0.824) in the development cohort and 0.692 (95% CI 0.568–0.801) in the external validation cohort. At the prevalence of moderate-or-greater AS observed in the external cohort (7.4%), this corresponds to an approximately 9.4-fold enrichment in precision relative to prevalence alone (Supplemental Figure 2).

**Supplemental Figure 2. Precision-Recall Curves for the eAS Model in the Development and External Validation Cohorts**

*Precision-recall curves show the relationship between recall (sensitivity) and precision (positive predictive value) for detecting moderate-or-greater AS. Left: development cohort (prevalence 4.5%). Right: external validation cohort (prevalence 7.4%). The shaded band represents the pointwise 95% bootstrap confidence interval; the dashed horizontal line indicates the precision expected under random classification at the observed prevalence. AP, average precision (area under the precision-recall curve).*

## **Decision Curve Analysis**

Decision curve analysis of the recalibrated model showed a net benefit exceeding both the treat-all and treat-none strategies across the range of threshold probabilities examined (0.0–0.8) in the external validation cohort (Supplemental Figure 3).

**Supplemental Figure 3. Decision Curve Analysis of the Recalibrated eAS Model in the External Validation Cohort**

*Decision curve analysis showing the net benefit of the recalibrated eAS model across threshold probabilities of 0.0–0.8 in the external validation cohort, compared with the "treat all" and "treat none" strategies. The model provided greater net benefit than either default strategy across nearly the entire threshold range shown.*

## **Alternative Classification Thresholds**

Applying classification thresholds selected in the development cohort to target 95% and 99% sensitivity, and fixing these thresholds for evaluation in the external validation cohort, yielded a sensitivity of 95.7% (specificity 85.9%) at the 95%-target threshold and a sensitivity of 100% (70/70; specificity 66.5%) at the 99%-target threshold, illustrating the sensitivity–specificity trade-off associated with selecting a higher-sensitivity operating point than the Youden-based threshold used in the primary analysis (Supplemental Table 15).

**Supplemental Table 15. Diagnostic Performance of the eAS Model at Alternative Classification Thresholds**

| **Threshold-selection**  **criterion** | **Cohort** | **Threshold** | **Sensitivity**  **(95% CI)** | **Specificity**  **(95% CI)** | **PPV**  **(95% CI)** | **NPV**  **(95% CI)** | **TP** | **TN** | **FP** | **FN** |
| --- | --- | --- | --- | --- | --- | --- | --- | --- | --- | --- |
| Youden Index  (primary analysis) | Development | 0.148 | 0.944  (0.903-0.979) | 0.888  (0.877-0.899) | 0.285  (0.246-0.327) | 0.997  (0.995-0.999) | 136 | 2693 | 341 | 8 |
|  | External | 0.148 | 0.929  (0.864-0.984) | 0.879  (0.855-0.900) | 0.378  (0.304-0.452) | 0.994  (0.987-0.999) | 65 | 774 | 107 | 5 |
| Target sensitivity ≥ 0.95 | Development | 0.116 | 0.951  (0.915-0.984) | 0.865  (0.853-0.877) | 0.250  (0.215-0.288) | 0.997  (0.995-0.999) | 137 | 2624 | 410 | 7 |
|  | External | 0.116 | 0.957  (0.902-1.000) | 0.859  (0.835-0.882) | 0.351  (0.286-0.417) | 0.996  (0.991-1.000) | 67 | 757 | 124 | 3 |
| Target sensitivity ≥ 0.99 | Development | 0.042 | 0.993  (0.977-1.000) | 0.640 (0.623-0.656) | 0.116  (0.099-0.135) | 0.999  (0.998-1.000) | 143 | 1942 | 1092 | 1 |
|  | External | 0.042 | 1.000  (1.000-1.000) | 0.665  (0.635-0.695) | 0.192  (0.153-0.232) | 1.000  (1.000-1.000) | 70 | 586 | 295 | 0 |

*This table presents the diagnostic performance of the eAS model when the classification threshold was selected to achieve a pre-specified target sensitivity (95% or 99%) in the development cohort, in addition to the pre-specified Youden index–based threshold (0.148) used in the primary analysis. As in the primary analysis, each threshold was determined using the development cohort only and then applied, unchanged, to the external validation cohort. Values are presented as estimate (95% bootstrap confidence interval; 2,000 resamples) unless otherwise noted.*

*Abbreviations: TP, true positive; TN, true negative; FP, false positive; FN, false negative; PPV, positive predictive value; NPV, negative predictive value.*

## **Projected Diagnostic Yield Across Assumed Prevalence Scenarios**

Projected diagnostic yield was calculated across a range of assumed AS prevalence values (1%, 3%, 5%, and the prevalence observed in this cohort, 7.4%). As expected, PPV decreased substantially at lower assumed prevalence (7.17% at 1% prevalence), whereas NPV remained above 99% across all prevalence scenarios examined (Supplemental Table 16).

**Supplemental Table 16. Observed and Projected Diagnostic Yield of the eAS Model per 1,000 individuals tested, Across AS Prevalence Scenarios**

|  | **Observed external cohort (7.4%)** | **Assumed Prev. 1%** | **Assumed Prev. 3%** | **Assumed Prev. 5%** |
| --- | --- | --- | --- | --- |
| TP | 68.4 | 9.3 | 27.9 | 46.4 |
| TN | 813.9 | 869.8 | 852.2 | 834.6 |
| FP | 112.5 | 120.2 | 117.8 | 115.4 |
| FN | 5.3 | 0.7 | 2.1 | 3.6 |
| PPV | 37.8% | 7.2% | 19.1% | 28.7% |
| NPV | 99.4% | 99.9% | 99.8% | 99.6% |

*This table projects the number of true-positive, true-negative, false-positive, and false-negative classifications per 1,000 individuals tested by the eAS model (using the pre-specified Youden index threshold of 0.148; sensitivity 92.9%, specificity 87.9%), together with the corresponding PPV and NPV, under the observed AS prevalence in the external validation cohort (7.4%) and three illustrative assumed prevalence scenarios (1%, 3%, 5%). These scenarios illustrate how PPV and NPV vary with disease prevalence and are not intended as precise epidemiological estimates, given substantial heterogeneity across published AS prevalence studies.*

*Abbreviations: TP, true positive; TN, true negative; FP, false positive; FN, false negative; PPV, positive predictive value; NPV, negative predictive value.*

## **False Negative Case Analysis**

**Supplemental Table 17. Detailed Clinical and Echocardiographic Profiles of False-Negative Cases in the External Validation Cohort.**

| **ID** | **Heart sound findings** | **Patient background** | **Other valvular disease** | **Hemodynamics** | **AS-related echo findings** | **Comment** |
| --- | --- | --- | --- | --- | --- | --- |
| 1 | Systolic murmur (±), accentuated S2 | 57, male, BMI 24.2 | AR moderate, MR mild, TR mild | BNP 20.4, EF 58%, SVi 53.4 mL/m² | Vmax 3.1 m/s, mean PG 23 mmHg, AVA 1.19 cm² | Follow-up for AS, no evidence of cardiac overload |
| 2 | Systolic murmur (+), accentuated S2 | 96, female | MR moderate, AR moderate, TR mild | BNP 205, EF 64.6% | Vmax 3.6 m/s, mean PG 26.8 mmHg, AVA 0.71 cm² | Severe AS with multiple concomitant valvular lesions; complex hemodynamics |
| 3 | Systolic murmur (+), not diminished S2 | 67, male, BMI 27.5 | None | BNP 29, EF 63%, E/e’ 15.4 | Vmax 3.2 m/s, mean PG 19.4 mmHg, AVA 1.14 cm² | Isolated moderate AS under follow-up; borderline mean PG may affect detection, no evidence of cardiac overload |
| 4 | Muffled Heart sounds, systolic murmur (-), diminished S2 | 86, male, BMI 19.3, history of AAA stent graft | AR mild, MR mild, TR mild | BNP 50.1, EF 62%, SVi 43 mL/m² | Vmax 2.5 m/s, mean PG 13.5 mmHg, AVA 1.13 cm² | Borderline mild–moderate AS under follow-up, no evidence of cardiac overload |
| 5 | Systolic murmur (±), accentuated S2 | 72, female, BMI 26.4, history of OMI post-CABG | AR mild, MR none, TR none | BNP 290 (eGFR 18.6), EF 54%, E/e’ 14.7 | Vmax 2.8 m/s, mean PG 20.6 mmHg, AVA 0.89 cm² | Borderline mild–moderate AS under follow-up |

*This table summarizes the clinical, phonocardiographic, and echocardiographic characteristics of patients with moderate-or-greater aortic stenosis (AS) who were misclassified as negative by the eAS model (false-negative cases) in the external validation cohort. Information includes heart sound findings, patient background, concomitant valvular diseases, hemodynamic parameters, AS-related echocardiographic measurements, and clinical follow-up comments. These cases illustrate potential factors contributing to false-negative predictions, such as borderline AS severity, concomitant valvular lesions, or complex hemodynamic conditions.*

## **Characterization of False-Positive Cases**

Compared with true-negative cases, false-positive cases were older (median age 78 vs. 72 years, P=0.002), more often female (54.2% vs. 31.9% female, P<0.001), and had higher BNP levels (P=0.001). False-positive cases also showed echocardiographic evidence of diastolic dysfunction, including a lower E/A ratio (P=0.008) and higher E/e′ ratio (P<0.001), and more severe values on all four key echocardiographic parameters used to define AS severity (Vmax, mean PG, AVA, and AVAI; all P<0.001). Notably, 21.5% of false-positive cases had a pre-existing diagnosis of mild AS, compared with 2.6% of true-negative cases (P<0.001), consistent with the discordant-AVA subgroup analysis described in Supplemental Table 12 (24 of 107 false positives, 22.4%). The distribution of aortic regurgitation severity also differed between groups (P=0.026), with a numerically higher proportion of moderate-or-greater aortic regurgitation among false positives (17.8% vs. 9.2%). Full characteristics are presented in Supplemental Table 18.

**Supplemental Table 18. Clinical and Echocardiographic Characteristics of False-Positive versus True-Negative Cases in the External Validation Cohort**

| Variable | FP (n=107) | TN (n=774) | P value |
| --- | --- | --- | --- |
| Age, median [IQR] | 78.0 [67.5–83.0] | 72.0 [63.0–80.0] | 0.002 |
| Male sex, n (%) | 49 (45.8) | 527 (68.1) | <0.001 |
| BMI, median [IQR] | 22.5 [20.6–25.6] | 23.2 [20.8–25.6] | 0.437 |
| Systolic BP, median [IQR] | 136 [120–152] | 135 [121–151] | 0.784 |
| Atrial fibrillation, n (%) | 10 (10.3) | 71 (10.3) | 1 |
| BNP, median [IQR] | 99 [42–261] | 53 [21–155] | 0.001 |
| LAVI, median [IQR] | 45.5 [30.0–59.8] | 38.0 [28.0–52.0] | 0.05 |
| LVDd, median [IQR] | 45.2 [41.0–52.0] | 47.0 [43.0–52.0] | 0.05 |
| LVDs, median [IQR] | 28.0 [24.0–35.4] | 30.0 [26.4–36.0] | 0.02 |
| LVEF (modified Simpson), median [IQR] | 59.5 [53.0–64.0] | 59.0 [49.0–63.0] | 0.186 |
| E/A ratio, median [IQR] | 0.72 [0.60–0.90] | 0.80 [0.64–1.10] | 0.008 |
| e′ septal, median [IQR] | 5.0 [3.9–6.3] | 5.1 [4.0–6.5] | 0.169 |
| e′ lateral, median [IQR] | 6.3 [4.7–7.7] | 6.3 [4.8–8.2] | 0.589 |
| E/e′ ratio, median [IQR] | 12.7 [10.1–17.0] | 10.8 [8.6–14.4] | <0.001 |
| AV Vmax, median [IQR] | 1.9 [1.4–2.3] | 1.3 [1.1–1.6] | <0.001 |
| AV mean PG, median [IQR] | 7.7 [4.8–11.6] | 3.8 [2.7–5.4] | <0.001 |
| AVA, median [IQR] | 1.7 [1.3–2.0] | 2.2 [1.8–2.6] | <0.001 |
| AVAI, median [IQR] | 1.1 [0.9–1.3] | 1.3 [1.1–1.6] | <0.001 |
| Pre-existing mild AS diagnosis, n (%) | 23 (21.5) | 20 (2.6) | <0.001 |
| AR severity, n (%) |  |  | 0.026 |
| None | 54 (50.5) | 453 (58.5) |  |
| Mild | 34 (31.8) | 250 (32.3) |  |
| Moderate | 16 (15.0) | 65 (8.4) |  |
| Severe | 3 (2.8) | 6 (0.8) |  |
| MR severity, n (%) |  |  | 0.476 |
| None | 36 (33.6) | 318 (41.1) |  |
| Mild | 50 (46.7) | 326 (42.2) |  |
| Moderate | 16 (15.0) | 104 (13.5) |  |
| Severe | 5 (4.7) | 25 (3.2) |  |
| TR severity, n (%) |  |  | 0.093 |
| None | 43 (40.2) | 398 (51.4) |  |
| Mild | 49 (45.8) | 309 (39.9) |  |
| Moderate | 10 ( 9.3) | 49 ( 6.3) |  |
| Severe | 5 ( 4.7) | 18 ( 2.3) |  |

Values are presented as median [IQR] or number (%). Continuous variables were compared using the Wilcoxon rank-sum test; categorical variables were compared using the chi-square test or Fisher's exact test, as appropriate.

Abbreviations: AR, aortic regurgitation; AS, aortic stenosis; AV, aortic valve; AVA, aortic valve area; AVAI, aortic valve area index; BMI, body mass index; BNP, B-type natriuretic peptide; BP, blood pressure; E/A, ratio of early to late (atrial) mitral inflow velocity; e′, mitral annular early diastolic velocity; E/e′, ratio of early mitral inflow velocity to mitral annular early diastolic velocity; FP, false positive; IQR, interquartile range; LAVI, left atrial volume index; LVDd, left ventricular end-diastolic diameter; LVDs, left ventricular end-systolic diameter; LVEF, left ventricular ejection fraction; mean PG, mean transvalvular pressure gradient; MR, mitral regurgitation; TN, true negative; TR, tricuspid regurgitation; Vmax, peak aortic valve velocity.

## **Qualitative Interpretability Analysis**

**Supplemental Figure 4. Grad-CAM-Enhanced Phonocardiographic Wavelet Representations and Clinical Characteristics of True- and False- Classified Cases**

*This figure presents representative phonocardiogram (PCG) wavelet images with Gradient-weighted Class Activation Mapping (Grad-CAM) overlays of the CNN model for each category of the confusion matrix: true positive, false positive, false negative, and true negative cases. For each example, patient demographics and key echocardiographic parameters related to aortic stenosis severity—including peak aortic valve velocity (Vmax), mean transvalvular pressure gradient (PG), and aortic valve area (AVA)—are shown.*

*The wavelet representations depict time–frequency characteristics of heart sounds, while Grad-CAM–derived attention maps are superimposed as red waveforms, indicating temporal regions contributed most strongly to the model’s prediction. Model attention was observed not only during mid systole, but also around the second heart (S2) and diastolic phases.*

*Arrows indicate systolic murmurs when visually identifiable. Comparison across cases highlights how subtle murmurs and complex acoustic patterns may influence model predictions.*

*Abbreviations: AS, aortic stenosis; BMI, body mass index; PG, transvalvular pressure gradient; AVA, aortic valve area; EF, ejection fraction; BNP, B-type natriuretic peptide.*

# **Supplemental References**

1.	Stassen J, Ewe SH, Singh GK, et al. Prevalence and Prognostic Implications of Discordant Grading and Flow-Gradient Patterns in Moderate Aortic Stenosis. *J Am Coll Cardiol*. 2022;80(7):666-676. doi:10.1016/j.jacc.2022.05.036

2.	Moura LM, Ramos SF, Pinto FJ, Barros IM, Rocha-Gonçalves F. Analysis of variability and reproducibility of echocardiography measurements in valvular aortic valve stenosis. *Rev Port Cardiol*. 2011;30(1):25-33.
